# Differential Interferon Signaling Regulation and Oxidative Stress Responses in the Cerebral Cortex and Cerebellum Could Account for the Spatiotemporal Pattern of Neurodegeneration in Niemann–Pick Disease Type C

**DOI:** 10.3390/genes15010101

**Published:** 2024-01-15

**Authors:** Andrew J. Tolan, Kayla L. Sanchez, Samuel D. Shin, Jacob B. White, Antonio Currais, David Soriano-Castell, Christopher G. Wilson, Pamela Maher, Salvador Soriano

**Affiliations:** 1 Department of Pathology and Human Anatomy, School of Medicine, Loma Linda University, Loma Linda, CA 92354, USAklsanchez@students.llu.edu (K.L.S.); dshin@students.llu.edu (S.D.S.); jwhite01@students.llu.edu (J.B.W.); 2 The Salk Institute for Biological Studies, 10010 North Torrey Pines Road, La Jolla, CA 92037, USA; acurrais@salk.edu (A.C.); dsorianocastell@salk.edu (D.S.-C.); 3 Lawrence D. Longo Center for Perinatal Biology, School of Medicine, Loma Linda University, Loma Linda, CA 92354, USA; cgwilson@llu.edu

**Keywords:** Niemann–Pick disease type C, neurodegeneration, interferon signaling

## Abstract

Niemann–Pick disease type C (NPC) is a fatal neurodegenerative condition caused by genetic mutations of the *NPC1* or *NPC2* genes that encode the NPC1 and NPC2 proteins, respectively, which are believed to be responsible for cholesterol efflux from late-endosomes/lysosomes. The pathogenic mechanisms that lead to neurodegeneration in NPC are not well understood. There are, however, well-defined spatiotemporal patterns of neurodegeneration that may provide insight into the pathogenic process. For example, the cerebellum is severely affected from early disease stages, compared with cerebral regions, which remain relatively spared until later stages. Using a genome-wide transcriptome analysis, we have recently identified an aberrant pattern of interferon activation in the cerebella of pre-symptomatic *Npc1^−/−^* mice. Here, we carried out a comparative transcriptomic analysis of cerebral cortices and cerebella of pre-symptomatic *Npc1^−/−^* mice and age-matched controls to identify differences that may help explain the pathological progression within the NPC brain. We report lower cerebral expression of genes within interferon signaling pathways, and significant differences in the regulation of oxidative stress, compared with the cerebellum. Our findings suggest that a delayed onset of interferon signaling, possibly linked to lower oxidative stress, may account for the slower onset of cerebral cortical pathology in the disease.

## 1. Introduction

Niemann–Pick disease type C (NPC) is a fatal neurodegenerative condition caused by genetic mutations of the *NPC1* (Chr. 18q11.2) or *NPC2* (Chr. 14q24.3) genes that encode the NPC1 and NPC2 proteins, respectively. While NPC affects all cell types in the body, its main feature is progressive neurodegeneration that results in premature death [1]. Upon the onset of neurologic symptoms, NPC progresses quickly, resulting in a dramatic shift from clinically normal development to severe and global neurologic deficits, including immobility and dementia [1]. Terminal-stage NPC patients require constant monitoring and life support. Thus, NPC patients and caregivers suffer from reduced quality of life, and most cases are inevitably fatal before reaching adulthood.

Gross NPC neuropathology displays well-defined spatiotemporal patterns of neurodegeneration. For example, the cerebellum is severely affected from early disease stages, with Purkinje neurons being particularly vulnerable [2,3], consistent with the cerebellar deficits common in NPC patients [2]. At the cellular and molecular levels, there are abnormalities of intracellular endolysosomal compartments, which also display accumulation of unesterified cholesterol, glycolipids, and sphingolipids [2,4,5]; abnormal calcium storage and signaling [5]; mitochondrial dysfunction; high concentrations of reactive oxygen species (ROS) and lipid peroxidation [6]; generation of damage-associated molecular patterns (DAMPs); and a highly pro-inflammatory environment that results from the activation of innate immune responses [7,8]. Neuroinflammation in the cerebellum is an early finding in the neurodegenerative cascade of NPC. In the most widely used *Npc1^−/−^* mice (BALB/c*^Nctr-Npc1miN/J^*), microglial activation and reactive astrocytosis have been reported as early as 2 weeks of age [9], prior to the typical onset of neurological deficits between 7 and 8 weeks of age observed in this strain. To explore the mechanisms that lead to early-stage neuroinflammation in the NPC cerebellum, our laboratory previously explored the inflammatory markers present in pre-symptomatic *Npc1^−/−^* mice [8]. Using a transcriptomic analysis, we demonstrated the presence of an atypical pattern of interferon signaling, involving both IFN-γ- and IFN-α-responsive genes, linked to microglial activation, anti-viral response, T-lymphocyte activation, and chemotaxis of T-lymphocytes, in pre-symptomatic *Npc1^−/−^* cerebella [8]. Biochemical analysis supported these findings, showing that (1) IP-10/CXCL10, a key regulator in interferon signaling, was the sole cytokine significantly upregulated in the *Npc1^−/−^* mouse cerebellum prior to disease onset, and (2) interferon-responsive pro-inflammatory markers were dramatically upregulated in the cerebella of terminal-stage *Npc1^−/−^* mice [8].

In stark contrast with the early and rapidly progressing neurodegeneration patterns in the NPC cerebellum, cerebral regions are relatively spared until later disease stages [10]. Here, we carried out a comparative transcriptomic analysis of cerebral cortices and cerebella of pre-symptomatic *Npc1^−/−^* mice and age-matched controls to search for clues that may help explain these spatiotemporal differences in the pathological progression within the NPC brain. Consistent with our previous work [8], we report the upregulation of genes within interferon signaling pathways in the cerebellum and further show that this pattern is not present in the cerebral cortex. In addition, we identified significant differences in the regulation of oxidative stress in the cerebral cortex compared with the cerebellum. Our findings suggest that a delayed onset of interferon signaling, possibly linked to lower oxidative stress, may account for the slower onset of cerebral cortical pathology in the disease.

## 2. Materials and Methods

### 2.1. Mice

*Npc1^−/−^* mice were generated from heterozygous mice of the strain BALB/c*^Nctr-Npc1miN/J^* from the Jackson Laboratory [8] The study was approved by the Loma Linda University Institutional Animal Care and Use Committee (LLU#8170041). Genotyping was conducted as described in reference [8].

### 2.2. Transcriptome Analysis

Mouse tissue was analyzed for transcriptomics as we have described [8]. Cerebral cortex samples from *Npc1^−/−^* and age-matched control mice were sent to GenUs (GenUs Biosystems, Northbrook, IL, USA) for analysis. RNA was extracted from samples and purified using Ribopure (Ambion; Fisher Healthcare, Houston, TX, USA) RNA isolation kit. Total RNA samples were quantitated with UV spectrophotometry (OD260/280). The quality of total RNA was then analyzed with an Agilent Bioanalyzer. After preparation of cDNA and cRNA strands, cRNA was hybridized to Agilent Mouse v2 GE 4 × 44 K arrays, and then scanned on an Agilent G2565 Microarray Scanner. Data were analyzed with Agilent Feature Extraction and GeneSpring GX v7.3.1 software [8].

### 2.3. Data Pre-Processing

Raw counts were normalized to average expression of all genes. Microsoft Excel files provided by GenUs were converted to comma-separated value (CSV) files, and then merged using the Python package Pandas (code in Supplementary Materials). Data were filtered to remove null values and duplicates. Data were then formatted in the form required for differential expression tools.

### 2.4. Differential Gene Expression Analysis

Pre-processed data were analyzed for differential gene expression. The statistical significance of normalized expression levels between groups was determined by Kruskal–Wallis one-way analysis of variation, followed by Welsh’s *t*-test between specific comparisons, with a *p*-value less than 0.05 considered significant. Log fold change was determined for each gene with the following comparisons: NPC cerebral cortex vs. NPC cerebellum, NPC cerebral cortex vs. wildtype cortex, NPC cerebral cortex vs. wildtype cerebellum, wildtype cerebral cortex vs. wildtype cerebellum, and NPC cerebellum vs. wildtype cerebellum.

### 2.5. Pathway Enrichment

Datasets with differentially expressed genes were imported into Ingenuity Pathway Analysis (IPA, Qiagen, Redwood City, CA, USA) and analyzed for a comprehensive look at differentially expressed pathways and ontologies. Full pathway enrichment data are included in the Supplemental Data. Core analysis was run, and pathways analyzed included neuroinflammation, cholesterol metabolism, toll-like receptor signaling, and autophagy. Within these groupings, pathways were filtered by their effect, such as pro-inflammatory or anti-inflammatory. Components not relevant to neurodegeneration, such as the effect of cancer drugs, were excluded from analysis.

Custom pathways were created based on KEGG (https://www.genome.jp/kegg/; accessed on 6 January 2024) and run through IPA to visualize gene expression within the pathways. Heatmaps were created to further visualize expression of each gene within specific pathways or with certain functions. Full resolution raw images of all figures are included in Appendix A.

### 2.6. Gene Set Enrichment Analysis

Gene set enrichment analysis was conducted using GSEA software (GSEA v4.3.2 Mac App; www.broadinstitute.org/gsea, accessed on 6 January 2024) [11] on significant differentially expressed genes. Gene set enrichment was run against Reactome interferon signaling, toll-like receptor 4 signaling cascade, and detoxification of reactive oxygen species gene sets as well as Biocarta nfkb pathway and Gene Ontology response to reactive oxygen species gene sets. Parameters used for GSEA were as follows: permutation type for enrichment analysis was based on phenotype, and the number of permutations was 1000. The chip type was “Mouse_Ensembl_Gene_ID_MSigDB.v2023.1.Mm.chip” for mapping of gene IDs. Gene sets with over 500 genes were excluded. Enrichment statistics were “weighted” and the metric for ranking genes was “Signal2Noise” with gene list sorting mode “real” and a “descending” method of gene list ordering. Normalized enrichment score (NES) and false discovery rate (FDR) were calculated by the GSEA, and FDR < 0.25 was considered significant, as recommended by the software developers. Heatmaps were created from the gene subsets responsible for the core enrichment of each gene set.

**Figure 1:**
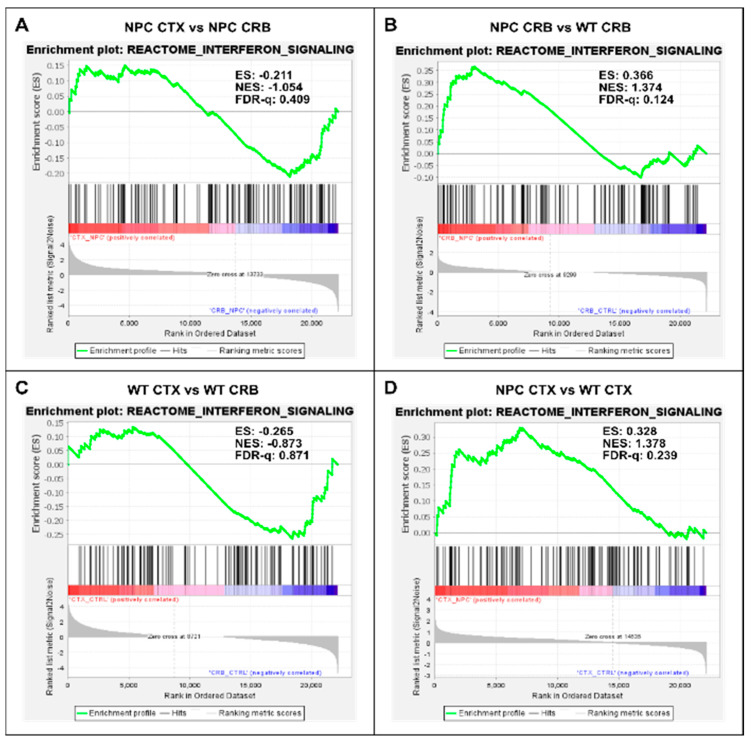
Gene set enrichment analysis reveals activation of the Interferon Signaling gene set in *Npc1^−/−^* mice. Interferon signaling is activated in cerebellum (CRB; (**B**)) and cerebral cortex (CTX; (**D**)) in *Npc1^−/−^* mice compared to wildtype controls (**B**,**D**). By contrast, there are no significant differences in enrichment between tissues in either genotype (**A**,**C**). ES = enrichment score; NES = normalized enrichment score; FDR-q = false discovery rate q value.

**Figure 2:**
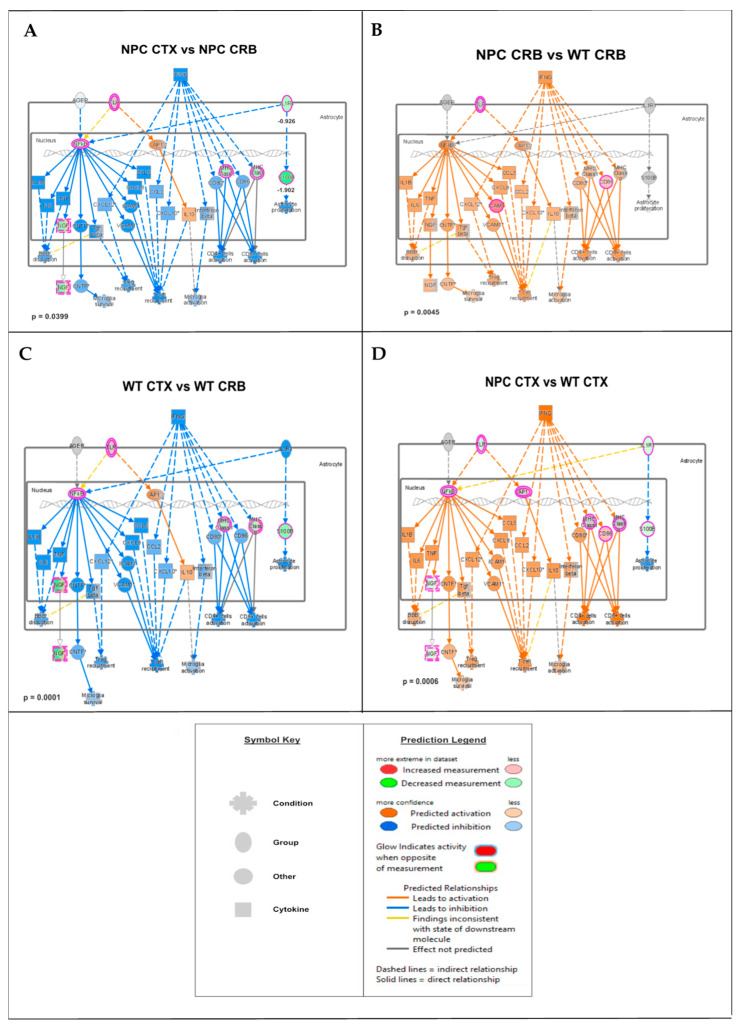
Merged network of IFN-γ- and IFN-α-responsive DEGs involved in microglial activation, anti-viral response, activation of T-lymphocytes, and chemotaxis of T-lymphocytes. (**A**,**C**) Interferon-mediated neuroinflammation pathways display lower levels in the cerebral cortex (CTX) when compared to the cerebellum (CRB), both in *Npc1^−/−^* (**A**) and wildtype mice (**C**). (**B**,**D**) The same pathways were upregulated in *Npc1^−/−^* mice, both in cerebellum, and cerebral cortex (**D**), indicating that, while pathway activation differences in these tissues are not disease-specific, they are upregulated in both tissues in NPC.

**Figure 3:**
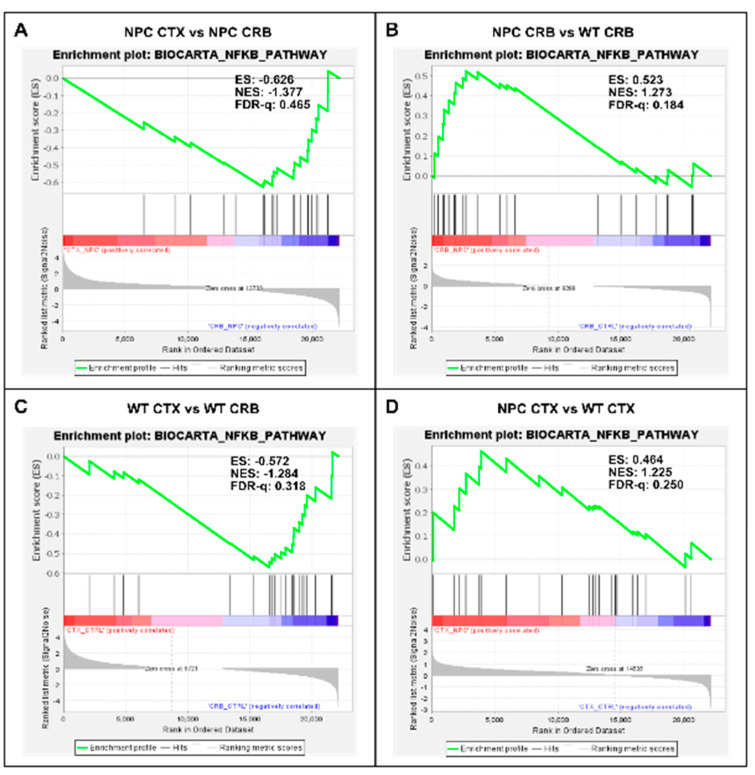
Gene set enrichment analysis of the Biocarta NF-kB pathway. Gene set enrichment analysis of the NF-kB signaling pathway shows no significant difference between tissues both in *Npc1^−/−^* mice and wildtype controls (**A**,**C**). Genotype comparison within tissues, on the other hand, shows that the NF-kB pathway gene set is significantly enriched in the cerebellum of *Npc1^−/−^* mice (**B**) but not in the cerebral cortex (**D**), compared to wildtype controls. ES = enrichment score; NES = normalized enrichment score; FDR-q = false discovery rate q value.

**Figure 4:**
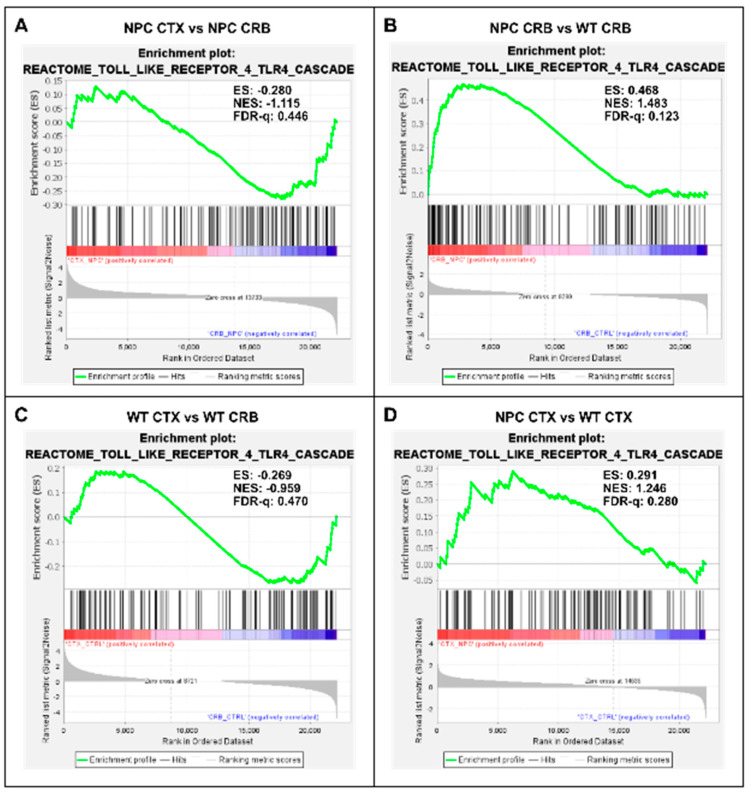
Gene set enrichment analysis of the Reactome Toll Like Receptor 4 (TLR4) pathway. Gene set enrichment analysis of the TLR4 signaling pathway shows no significant difference between tissues in *Npc1^−/−^* mice (**A**) or in wild-type controls (**C**). Notably, while TLR4 pathway gene set enrichment is significantly higher in the cerebellum of *Npc1^−/−^* mice compared to the cerebral cortex (**B**), no differences were found between tissues in the wildtype brain (**D**). ES = enrichment score; NES = normalized enrichment score; FDR-q = false discovery rate q value.

**Figure 5:**
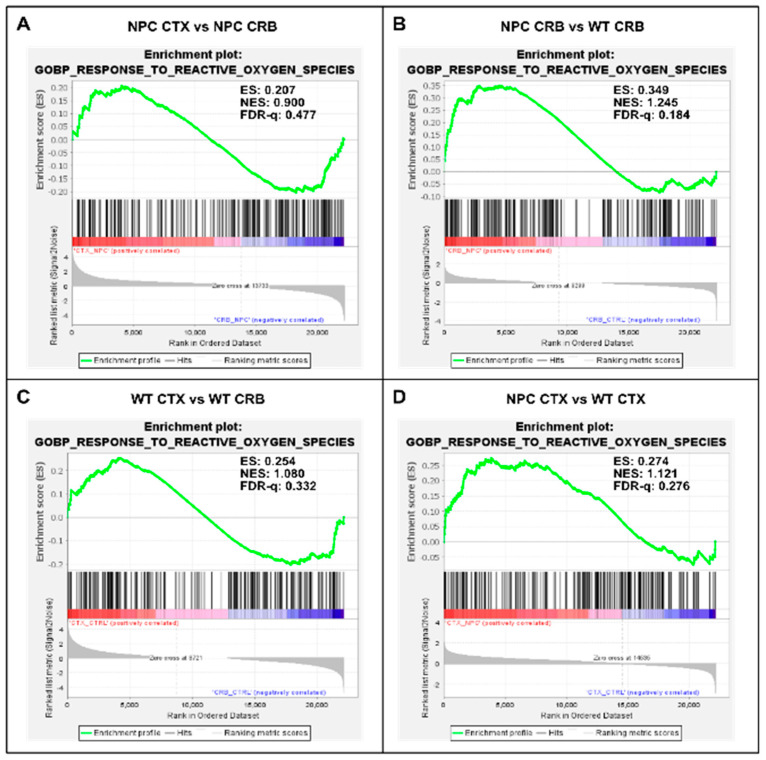
Gene set enrichment analysis of GOPB Response to Oxygen Species. (**A**,**C**). Response to Oxygen Species is not enriched in cerebellum versus cerebral cortex in *Npc1^−/−^* mice (**A**) and wildtype controls (**C**). (**B**,**D**). Comparisons between *Npc1^−/−^* and wildtype mice show increased activation in the cerebellum (**B**), but not in the cerebral cortex (**D**). ES = enrichment score; NES = normalized enrichment score; FDR-q = False discovery rate q-value.(**B**,**D**).

**Figure 6:**
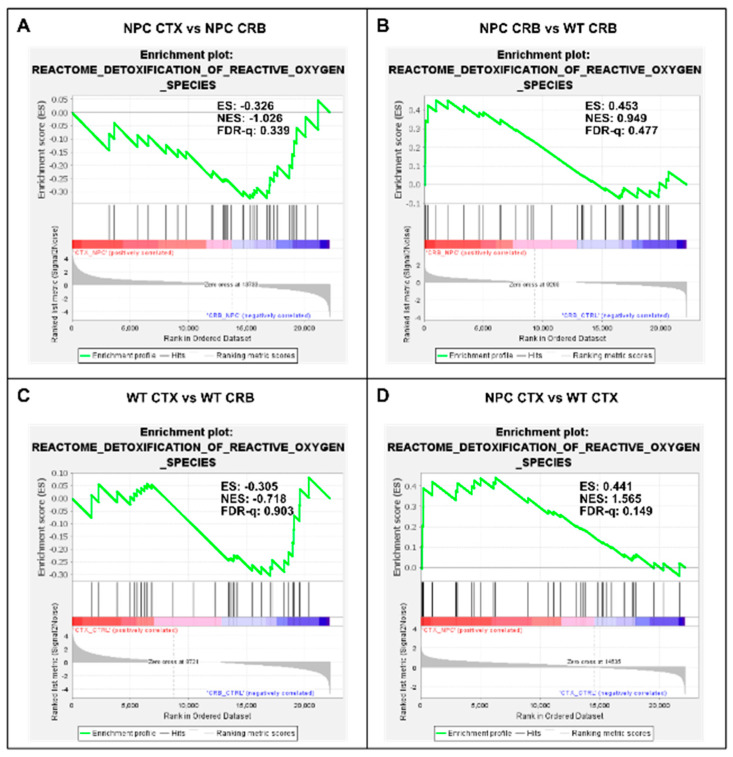
Gene set enrichment analysis of the Reactome Detoxification of Reactive Oxygen Species pathway. (**A**,**C**) Detoxification of Reactive Oxygen Species is not enriched in cerebellum versus ccerebral cortex in presymptomatic *Npc1^−/−^* mice (**A**) and wildtype controls (**C**). (**B**,**D**) Comparisons between *Npc1^−/−^* and wildtype mice show gene set enrichment in the cerebral cortex of *Npc1^−/−^* mice (**D**) but not in the cerebellum (**B**) ES = enrichment score; NES = normalized enrichment score; FDR-q = False discovery rate q-value.

High-resolution versions of gene set enrichment analyses presented in Figure 1, Figure 2, Figure 3, Figure 4, Figure 5 and Figure 6, together with heatmaps for each group comparison, are presented in Appendix A.

## 3. Results

### 3.1. Interferon Signaling Is Higher in the Cerebellum Than in the Cerebral Cortex in NPC

We have previously shown that IFN-α and IFN-γ signaling are exacerbated in the NPC cerebellum at 3 weeks of age, prior to disease onset [8]. Functional analyses of the differentially expressed genes revealed that IFN-γ- and IFN-α-responsive genes are involved in microglial activation, anti-viral response, T-lymphocyte activation, and T-lymphocyte chemotaxis pathways [8]. Consequently, we hypothesized that this untimely, IFN-driven activation of pro-inflammatory pathways may be an early driver of pathogenesis in NPC, contributing to the progressive loss of cerebellar Purkinje neurons and the early onset of cerebellar symptoms [8]. However, it is not clear whether early IFN-driven inflammation is restricted to the cerebellum at this early stage, or if it extends to regions of the brain that are less vulnerable, but ultimately also affected by NPC disease [10]. Here, we addressed that question with a comparative analysis of IFN-driven inflammation based on the examination of cerebral cortex and cerebellum transcriptomes from pre-symptomatic *Npc1^−/−^* mice and age-matched wildtype mice.

Our initial analysis showed that, in the *Npc1^−/−^* cerebral cortex, microarray hybridization and transcriptome analysis identified 321 differentially expressed genes (DEGs) versus *Npc1^−/−^* cerebellum, of which 199 were upregulated and 122 were downregulated (Supplementary Table S1). In comparison, 387 DEGs were identified in *Npc1^−/−^* cerebella versus wildtype cerebella, of which 176 were upregulated and 211 were downregulated Supplementary Table S2 in reference [8].

GSEA analysis indicated the activation of immune-related gene sets in the *Npc1^−/−^* cerebral cortex versus wildtype controls using the H1-Hallmark database (Supplemental Table S2). IPA upstream analysis further highlighted that IFN-γ is highly upregulated in the cerebral cortex of *Npc1^−/−^* mice, being in fact the top activated molecule in the upstream master regulator prediction analysis, with 84 related genes and an activation Z-score of 4.104 (Table 1). M-CSF/CSF1 and GM-CSF/CSF2 were two other major cytokine master regulators identified in this analysis (Table 1).

Of the 84 IFN-γ-related DEGs, 28 genes also co-mapped with IFN-α (Supplementary Figure S1). Functionally, IPA disease and function analysis identified differential expression of 15 genes related to microglial activation, 42 genes related to activation of antigen-presenting cells, 31 genes related to anti-microbial response, and 37 genes related to activation of T-lymphocytes (Supplementary Figure S2). Anti-viral response as a functional gene group was not found to be enriched in the IPA analysis of the *Npc1^−/−^* cortical transcriptome. This is a key difference with our recent findings showing that 15 IFN-γ-responsive DEGs from the pre-symptomatic *Npc1^−/−^* cerebella are involved in anti-viral response Figure 3 in reference [8].

In addition to this difference in anti-viral response activation between the cerebral cortex and cerebellum in the pre-symptomatic *Npc1^−/−^* brain, there was also a clear difference in the transcript expression of various pro-inflammatory cytokines between the two brain regions (Supplementary Table S3). In the *Npc1^−/−^* mice, Ccl2, Ccl5, Ccl6, Ccl7, Cxcl6, and Cxcl10 were significantly upregulated in the cerebellum while Ccl4, Ccl9, Ccl21, Cklf, Csf1, Cxcl3, Cxcl12, Ebi3, Il9, and Spred2 were significantly upregulated in the cerebral cortex (Supplementary Table S3). Notably, IP-10/Cxcl10 and Rantes/Ccl5, both of which are IFN-γ-responsive and are linked to all four major inflammatory pathways identified in pre-symptomatic *Npc1^−/−^* cerebella, are upregulated in this brain region but not in the cerebral cortex.

By contrast, further analysis of interferon differences between the cerebral cortex and cerebellum using GSEA of the reactome interferon signaling gene set (Figure 1) showed no differences between the cerebral cortex and cerebellum, as defined by an FDR-q < 0.25, both in *Npc1^−/−^* (Figure 1A; FDR-q = 0.409) and wildtype (Figure 1C; FDR-q = 0.871) mice. However, *Npc1^−/−^* mice showed a significant increase in interferon signaling versus wildtype controls in the cerebellum, (FDR-q = 0.124; Figure 1B and reference [8]) and, to a more modest extent, in the cerebral cortex (Figure 1D; FDR-q = 0.239).

These findings are consistent with the functional analyses described in Supplementary Figures S1 and S2, showing significant differences in IFN pathway regulation between the cerebral cortex of *Npc1^−/−^* mice and those of wildtype controls. Next, we carried out further IPA functional analysis comparisons of the brain regions and genotypes that might unveil differences in these pathways that would not be observed by GSEA, which ranks the genes within a set based on differential expression, generating an enrichment score that indicates whether the genes are clustered towards the beginning or end of the ranked list, without providing information on how those genes interact within biological pathways. As shown in Figure 2, while the cerebellum presents higher activation levels of the IFN-γ and IFN-α pathways in *Npc1^−/−^* mice than wildtype controls (Figure 2B; NPC CRB vs. WT CRB), as we have previously described [8], the cerebral cortex presents lower levels of these pathways than in the cerebellum in *Npc1^−/−^* mice (Figure 2A; NPC CTX vs. NPC CRB), leading to predicted lower levels of T-cell recruitment, microglial activation, and microglial survival (Figure 2A). Interestingly, this difference in pathway activation is mirrored in wildtype mice (Figure 2C), and IFN-linked signaling is higher in both tissues in the *Npc1^−/−^* brain versus wildtype controls (Figure 2B,D).

### 3.2. Inflammatory Pathways Linked to DAMP Generation, Excessive ROS, and Lipid Peroxidation in the Npc1^−/−^ Brain Are More Active in the Cerebellum

Our findings so far indicate that baseline interferon-mediated neuroinflammation is more active in the cerebellum than in the cerebral cortex of wildtype mice, and it is exacerbated in both tissues in the *Npc1^−/−^* brain. Next, we searched for upstream changes that could potentially contribute to this IFN-linked activation. While interferons display pleiotropic effects that drive both innate and adaptive immune responses [12,13], in the context of NPC, we asked whether the presence of damage-associated molecular patterns (DAMPs), which are prominent in NPC and are associated with mitochondrial and endolysosomal membrane damage, increased generation of reactive oxygen species (ROS), and lipid peroxidation [14,15,16,17], might be a significant source of IFN-linked activity in the pre-symptomatic NPC brain. Specifically, we asked whether NF-κB signaling and toll-like receptor 4 (TLR4) cascade pathways are exacerbated in the *Npc1^−/−^* cerebellum. NF-κB and TLR4 can be activated by DAMPs [14,15], and TLR4, which is present in the aberrantly enlarged endosomal compartment in NPC, mediates inflammatory cytokine expression via NFκB [14,15,18,19]. As shown in Figure 3, the gene set associated with NF-κB signaling is not enriched in the cerebellum versus the cerebral cortex in *Npc1^−/−^* (Figure 3A; FDR-q = 0.465) or wildtype brains (Figure 3C; FDR-q = 0.318). When evaluating differences within tissues in both genotypes, the NF-κB signaling gene set was enriched in the *Npc1^−/−^* cerebellum versus wildtype mice (Figure 3B; FDR-q = 0.184), but not in the cerebral cortex (Figure 3D; FDR-q = 0.25), consistent with the notion that NF-κB signaling upregulation may be confined to the cerebellum in the *Npc1^−/−^* brain.

Gene set enrichment analysis of the TLR4 cascade showed no enrichment between tissues in either *Npc1^−/−^* or wildtype brains (Figure 4A,C; FDR-q = 0.446, 0.470 respectively). Analysis of the TLR4 gene set showed significant enrichment in the cerebellum of *Npc1^−/−^* mice compared to wildtype mice (Figure 4B; FDR-q = 0.123) but not in the cerebral cortex (Figure 4D; FDR-q = 0.28). Taken together, these findings indicate that the gene sets involved in NF-kB and TLR4 signaling are significantly enriched in the *Npc1^−/−^* cerebellum compared to wildtype controls, which is consistent with the exacerbated interferon signaling observed in the cerebella of pre-symptomatic *Npc1^−/−^* mice.

Because the excess DAMP generation leading to high levels of ROS and lipid peroxidation in the *Npc1^−/−^* brain [14,15,16,17] would be expected to induce an antioxidant response, we carried out enrichment analyses of the Gene Ontology Biological Process Response to Reactive Oxygen Species gene set, as well as the Reactome Detoxification of Reactive Oxygen Species gene set, to measure relative differences in the activation of responses to ROS in *Npc1^−/−^* and wildtype mice. The former gene set highlights the presence of a wide range of functional changes as a result of a reactive oxygen species stimulus, such as the predicted oxidative environment in the *Npc1^−/−^* cerebellum (https://www.gsea-msigdb.org/gsea/msigdb/mouse/geneset/GOBP_RESPONSE_TO_REACTIVE_OXYGEN_SPECIES.html; accessed on 6 January 2024). By contrast, the Detoxification of Reactive Oxygen Species gene set includes genes corresponding to detoxifying enzymes, such as different glutathione peroxidases and superoxide dismutases (https://www.gsea-msigdb.org/gsea/msigdb/mouse/geneset/REACTOME_DETOXIFICATION_OF_REACTIVE_OXYGEN_SPECIES.html; accessed on 6 January 2024). As shown in Figure 5, the Response to Reactive Oxygen Species gene set was not different between the cerebral cortex and cerebellum, either in *Npc1^−/−^* mice (Figure 5A; FDR-q = 0.477) or in wildtype controls (Figure 5C; FDR-q = 0.332). Comparisons between genotypes revealed gene set enrichment in the cerebellum of *Npc1^−/−^* mice versus wildtype controls (Figure 5B; FDR-q = 0.184) but not in the cerebral cortex (Figure 5D; FDR-q = 0.276), which is consistent with the presence of higher ROS levels in the cerebellum of *Npc1^−/−^* mice. On the other hand, GSEA of the Reactome Detoxification of Reactive Oxygen Species set revealed enrichment only in the cerebral cortex of *Npc1^−/−^* mice compared to wildtype controls (Figure 6D; FDR-q = 0.149), suggesting an impaired detoxifying response to the oxidative stress environment of the NPC cerebellum.

## 4. Discussion

One major question in the natural history, clinical presentation, and neuropathology of NPC is the differential susceptibility of the brain’s anatomical regions to the pathologic onset and progression of NPC neurodegeneration. For example, cerebellar symptoms are one of the first neurologic symptoms present in human NPC patients [1,20,21], and this pathology is directly correlated with the severe loss of cerebellar Purkinje neurons in NPC [22,23]. On the other hand, cortical symptoms, such as memory deficits, appear relatively later in the natural history of NPC [1,20,21], and this cerebral cortical sparing is also paralleled in the neuropathology at the cellular level [2,10,24].

We have recently utilized a genome-wide transcriptome analysis of the initially-affected NPC cerebellum at a pre-symptomatic stage to investigate early mechanisms of pathogenesis. We reported evidence of an atypical pattern of interferon signaling that involves both IFN-γ- and IFN-α-responsive genes in pre-symptomatic *Npc1^−/−^* mice [8]. These findings led us to hypothesize that aberrant IFN activation could be a key driver of disease pathogenesis. If this hypothesis were correct, it would be reasonable to expect increased activation of inflammatory responses in the more susceptible cerebellum, in comparison to cerebral cortical brain regions that, as mentioned above, are relatively spared by the neurodegeneration. To test that hypothesis, we used a transcriptomic analysis to compare IFN signaling activation in the cerebellum and cerebral cortex of pre-symptomatic *Npc1^−/−^* mice and wildtype controls. We reasoned that these comparative analyses of the cerebellum and cerebral cortex, which are affected in early and late NPC, respectively, could help identify differential mechanisms of pathogenesis.

We found gene set enrichment of the interferon signaling pathway in the cerebellum of *Npc1^−/−^* mice as compared to wildtype controls (Figure 1B), and IPA analysis further revealed differences in the regulation of IFN pathways between the cerebellum and cortex (Supplementary Figures S2 and S3 and Figure 2). Of particular interest is the lack of anti-viral response activation in the cerebral cortex, which is upregulated in the cerebellum [8]. This difference may have pathogenic relevance, given that the NPC1 protein is directly involved in the pathogenesis of viral infection [25,26], and that, in viral infection, IFN-γ is a key player in the activation of the adaptive immune response against intracellular pathogens [27]. Furthermore, a defect in the IFN-γ-downstream IP-10/CXCL10 cytokine results in refractory viral infections [28]. The latter finding is of particular relevance here, because IP-10/CXCL10 is the major cytokine transcript identified in the *Npc1^−/−^* cerebellum (11.722-fold up), but it is not upregulated in the cerebral cortex (Supplementary Table S3). Therefore, it is plausible that the higher activation of the anti-viral function pathway in the cerebellum may lead to enhanced IP-10/CXCL10-dependent inflammation, thereby accelerating the pathogenic process in this area of the brain.

Regarding a potential upstream cause of this early inflammation pattern in the cerebellum, we reasoned that the widespread presence of DAMPs, excessive generation of ROS, and lipid peroxidation reported in NPC, which are associated with membrane damage at the mitochondria and the aberrantly enlarged endolysosomal compartments [14,15,16,17,18], could be triggering sterile inflammation patterns through NF-kB and toll-like receptor signaling pathways, leading to interferon signaling. Our findings are consistent with that notion, showing that the gene sets for both pathways were enriched in the cerebellum in the *Npc1^−/−^* brain, but not in the cerebral cortex (Figure 3B and Figure 4B), mirroring the enrichment pattern of the interferon signaling gene set (Figure 1B; [8]).

Given the oxidative stress environment initiated by the widespread presence of DAMPs and an excessive generation of ROS and lipid peroxidation in the NPC brain [14,15,16,17,18], we were interested to see whether there was a differential activation of antioxidant response activity in *Npc1^−/−^* mice. We reasoned that an impaired antioxidant response in the *Npc1^−/−^* brain could plausibly contribute to the inflammatory activation initiated in response to such a pro-oxidative environment. Analyses of Response to Oxygen Species and Detoxification of Reactive Oxygen Species gene sets showed results consistent with that notion. As shown in Figure 5B, the former gene set was only enriched in the *Npc1^−/−^* cerebellum, a pattern similar to those seen for inflammation markers in Figure 1B, Figure 3B and Figure 4B. By contrast, analysis of the Detoxification of Reactive Oxygen Species gene set, which reflects the expression levels of a wide range of detoxifying enzymes against oxidative stress, showed no enrichment in the *Npc1^−/−^* cerebellum, which would be consistent with the enhanced oxidative stress environment in this tissue [14,15,16,17,18]. Notably, we observed enrichment of this gene set in the cerebral cortex of *Npc1^−/−^* mice, suggesting the possibility that activation of detoxifying pathways may contribute to the slower inflammatory activation and subsequent pathogenic onset and progression in this tissue. Future work will determine whether an enhanced protective response in the cerebral cortex of *Npc1^−/−^* mice correlates with a lower pro-oxidative environment and a relative inhibition of the immune responses in this tissue. 

## 5. Conclusions

**Figure 7:**
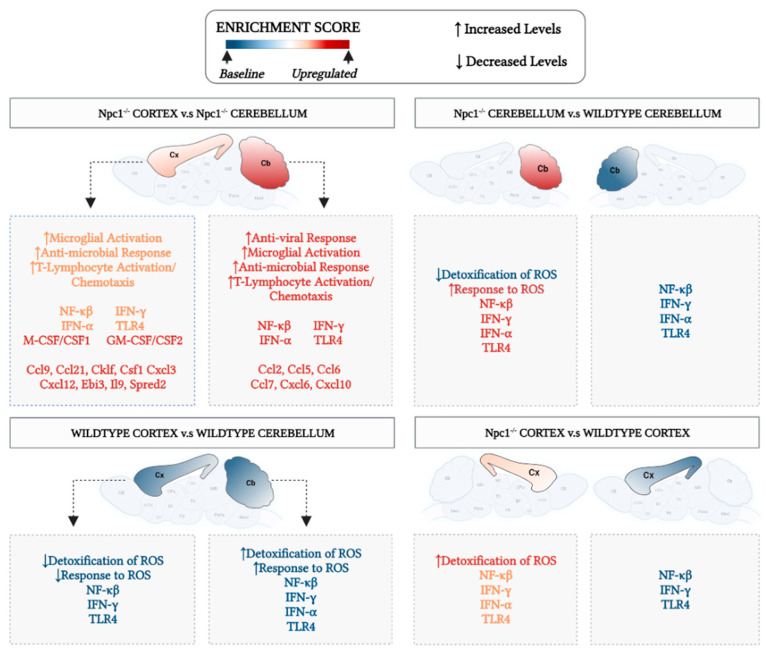
Different patterns of inflammatory gene activation in cerebral cortex and cerebellum of *Npc1^−/−^* mice. The enrichment score in the central circle of the figure represents arbitrary-color-coded relative changes at multiple levels of evaluation: cytokines and chemokines; gene sets; biological pathways, and phenotype severity in cerebral cortex (Cx) and cerebellum (Cb). All comparisons are described clockwise from the top. *Npc1^−/−^* Cortex vs. *Npc1^−/−^* cerebellum. The most severe disease phenotype is observed in the cerebellum of *Npc1^−/−^* mice (red-colored Cb), and it correlates with the highest enrichment of NF-kB, TLR4, IFN-g and IFN-a gene sets and the highest upregulation of anti-viral response, microglial activation, anti-microbial response, and T-lymphocyte activation/chemotaxis, compared to the cerebral cortex. The enrichment of the Detoxification/Response to ROS gene sets show the opposite pattern, being higher in Cx than in Cb. Thus, it is plausible that a combination of a weaker oxidative stress response and stronger inflammation activation in the cerebellum could explain, at least in part, the earlier and more severe onset and progression of pathogenesis in this area of the brain. *Npc1^−/−^* cerebellum vs wildtype cerebellum. All parameters for wildtype cerebellum are shown in blue to denote baseline levels. As highlighted above, the combination of a relatively low enrichment in Detoxification/Response to ROS pathways and high levels of inflammatory parameters contribute to an enhanced vulnerability in the cerebellum. Wildtype cerebral cortex vs wildtype cerebellum. All parameters are depicted in blue color to denote base values in both Cb and Cx in the wildtype brain. *Npc1^−/−^* cerebral cortex vs wildtype cerebral cortex. While there is a relative increase of inflammatory markers (shown in orange), the enrichment patterns of Detoxification/Response to ROS gene sets suggest a mechanism for a slower inflammatory activation and pathogenic onset and progression in the cerebral cortex (Cx depicted in orange).

Figure 7 summarizes our findings and highlights our key conclusions from those findings, namely the presence of different patterns of inflammatory gene activation in the cerebral cortex and cerebellum in *Npc1^−/−^* mice, and their correlation with different protective responses against ROS, providing a working model to explore the possibility that enhanced protection against ROS in the cerebral cortex reduces inflammation, and delays disease onset and progression in this area of the brain.

## Figures and Tables

**Table 1:** Top eight predicted cytokine/chemokine upstream regulators of the DEGs identified in the *Npc1^−/−^* mouse cerebral cortex transcriptome, as determined by IPA upstream analysis. # target = number of downstream gene targets (DEGs) of each upstream regulator.

Upstream Regulator	*Npc1^−/−^* vs. *Npc1^+/+^*		
	Z-Score	*p*-Overlap	# Target
IFN-γ	4.104	5.12 × 10^−11^	84
M-CSF/CSF1	3.785	6.17 × 10^−6^	21
GM-CSF/CSF2	3.598	1.00 × 10^−5^	33
IL-33	3.036	1.22 × 10^−3^	17
TNFα	2.908	9.37 × 10^−9^	96
IL-4	2.902	1.68 × 10^−6^	57
IL-6	2.737	4.26 × 10^−6^	46
IL-3	2.460	3.66 × 10^−3^	21

**Figure A1:**
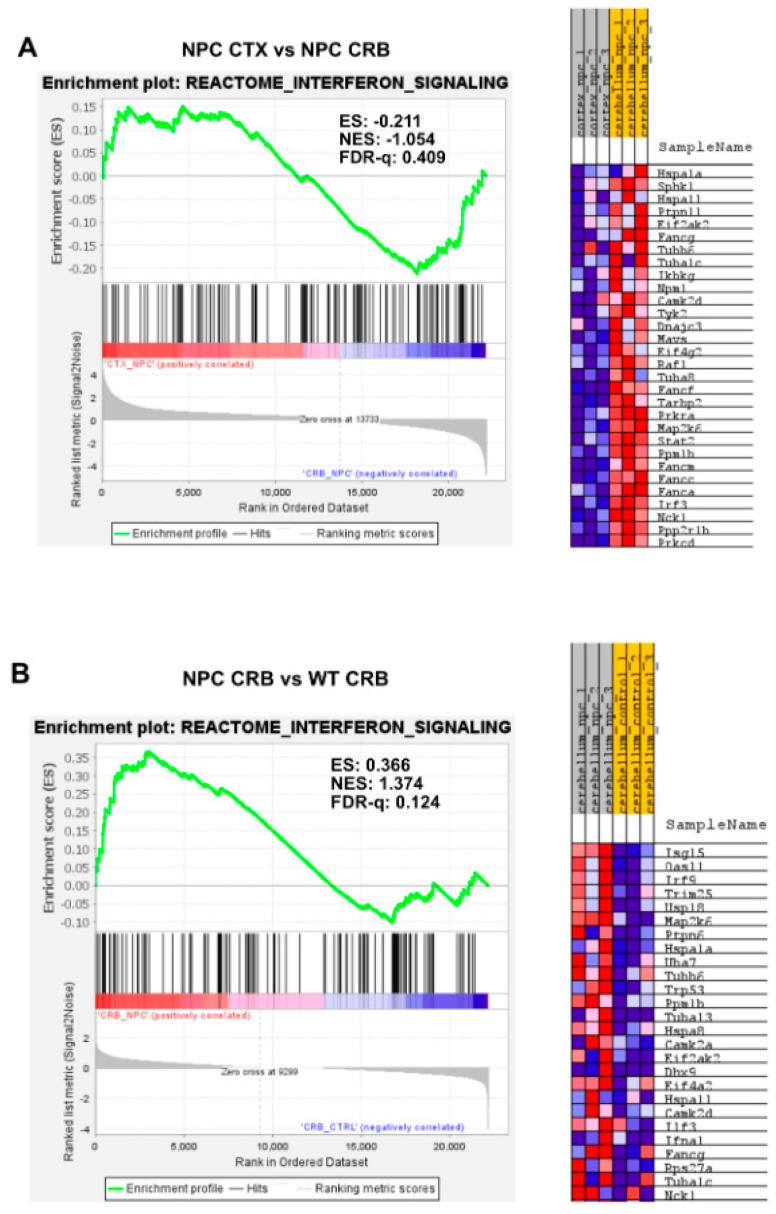

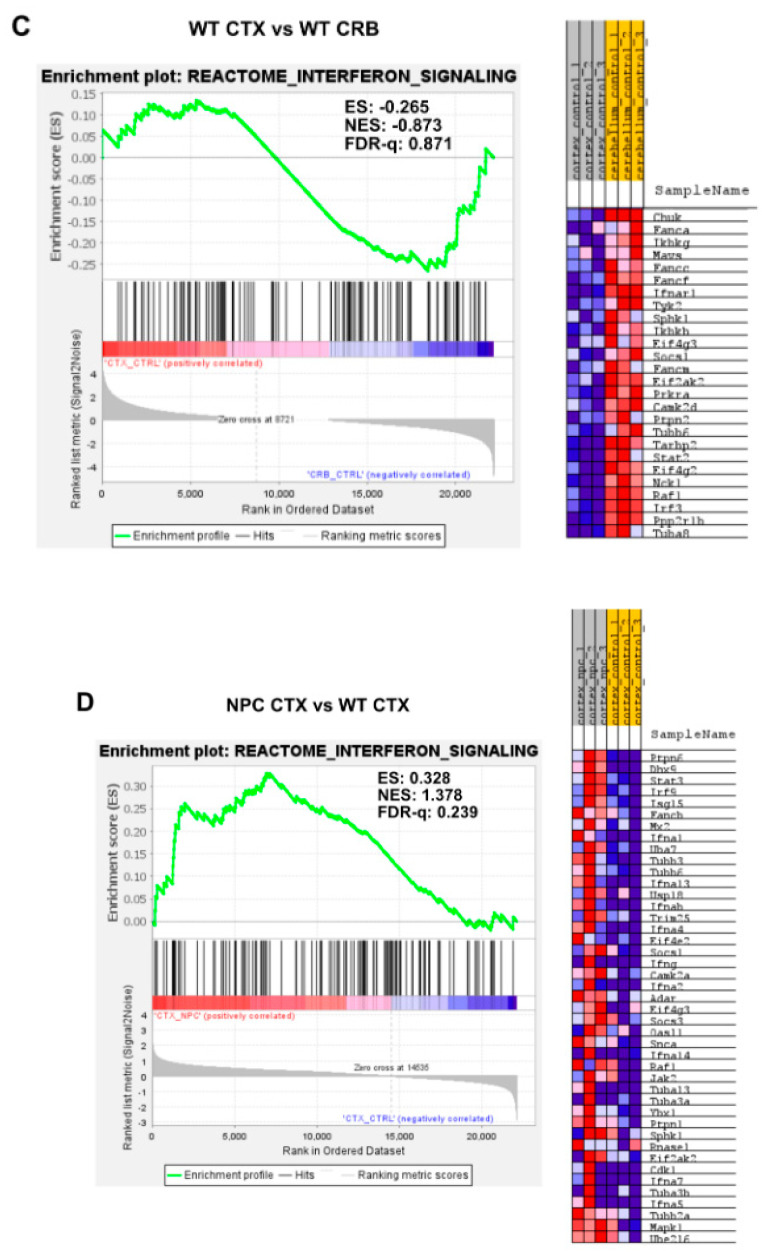
(**A**) Expanded version of Figure 1A from the main text with additional heatmap of core enriched genes. (**B**) Expanded version of Figure 1B from the main text with additional heatmap of core enriched genes. (**C**) Expanded version of Figure 1C from the main text with additional heatmap of core enriched genes. (**D**) Expanded version of Figure 1D from the main text with additional heatmap of core enriched genes.

**Figure A2:**
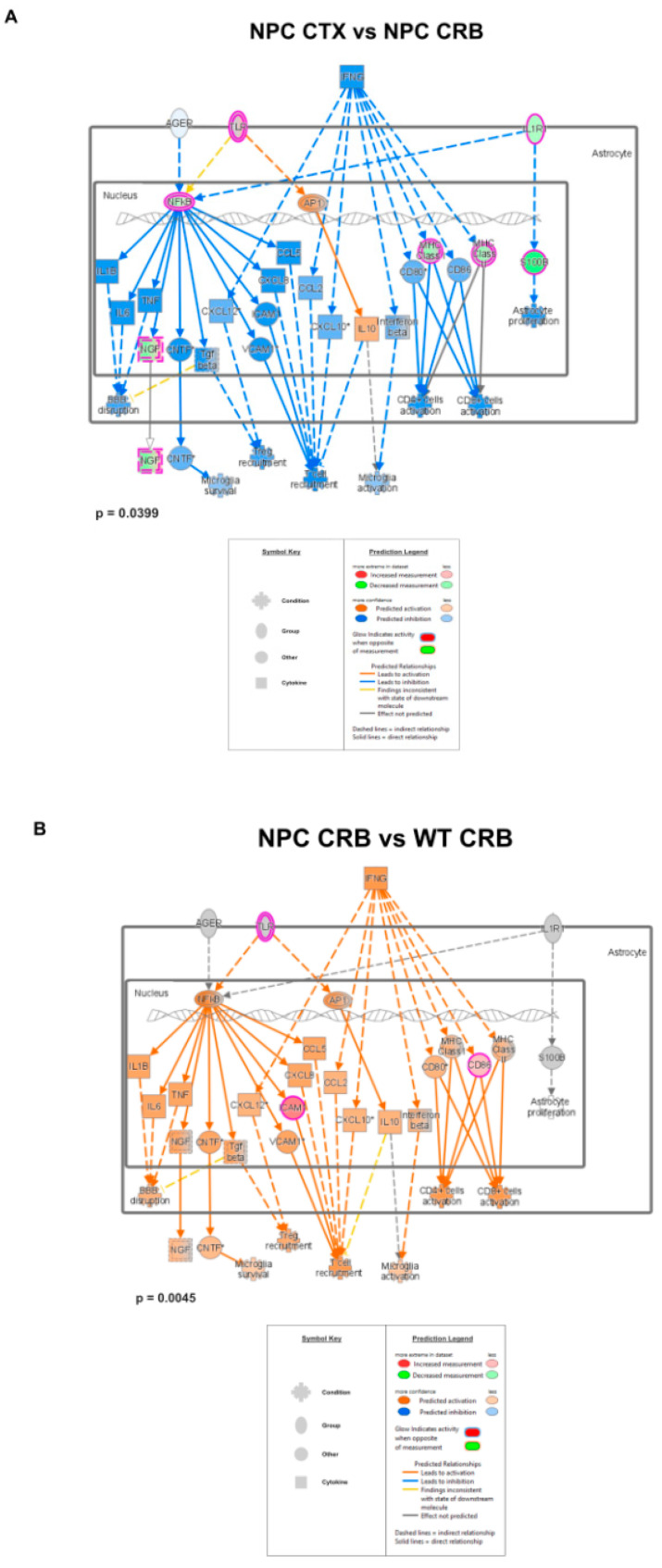

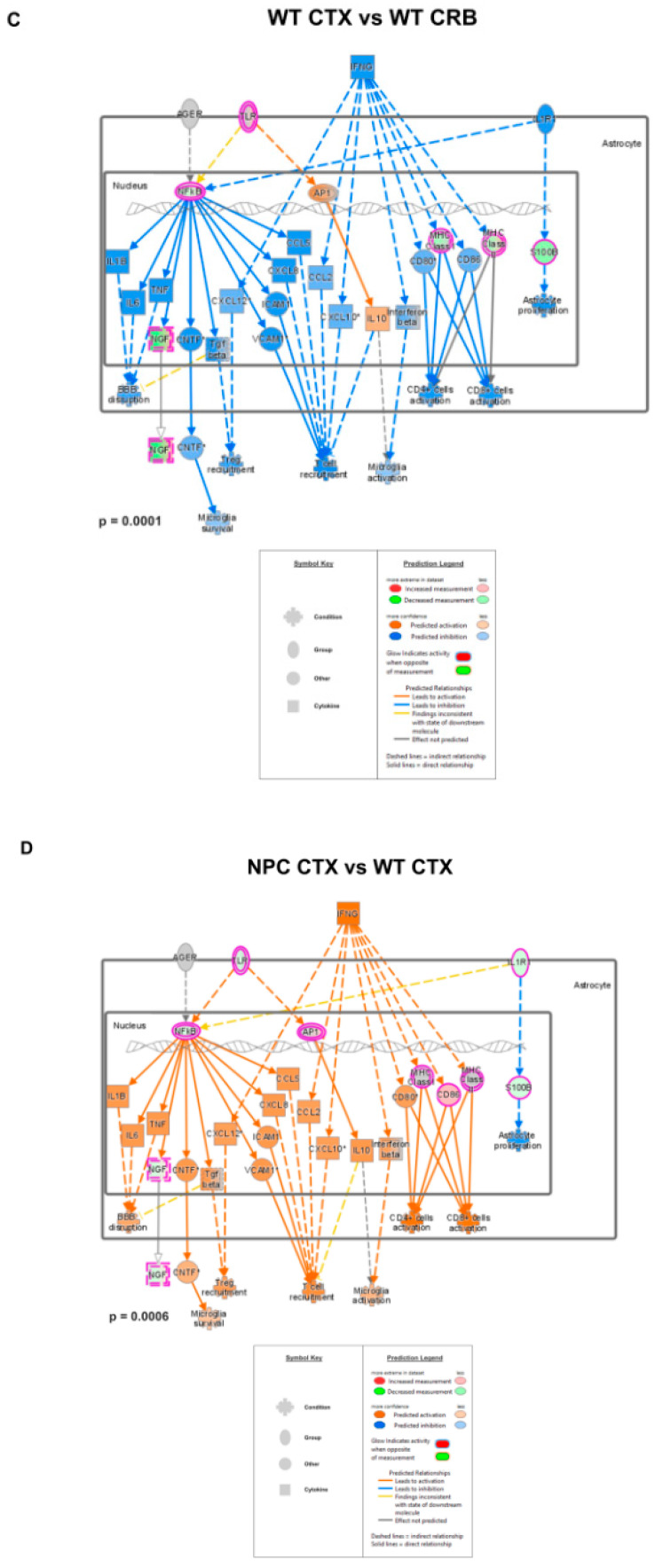
(**A**) Expanded version of Figure 2A from the main text to enhance readability of individual genes in the pathway. (**B**) Expanded version of Figure 2B from the main text to enhance readability of individual genes in the pathway. (**C**) Expanded version of Figure 2C from the main text to enhance readability of individual genes in the pathway. (**D**) Expanded version of Figure 2D from the main text to enhance readability of individual genes in the pathway.

**Figure A3:**
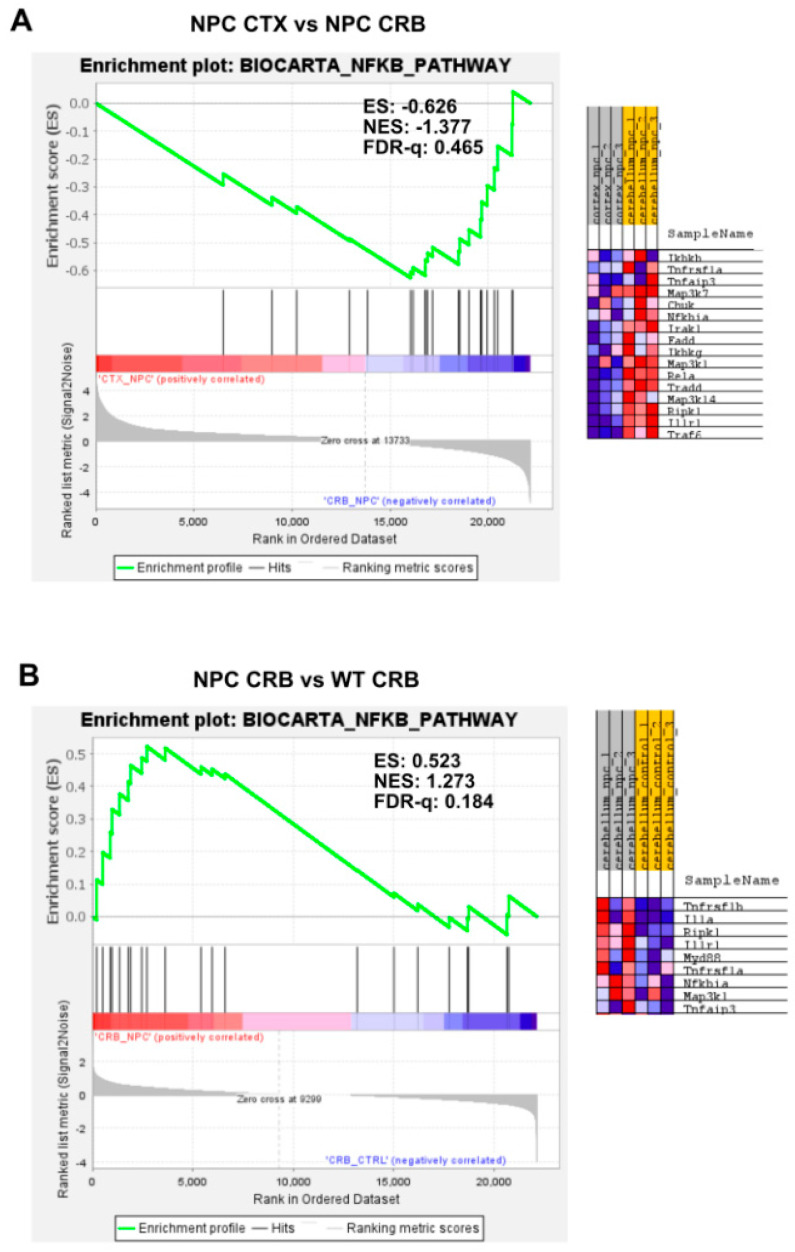

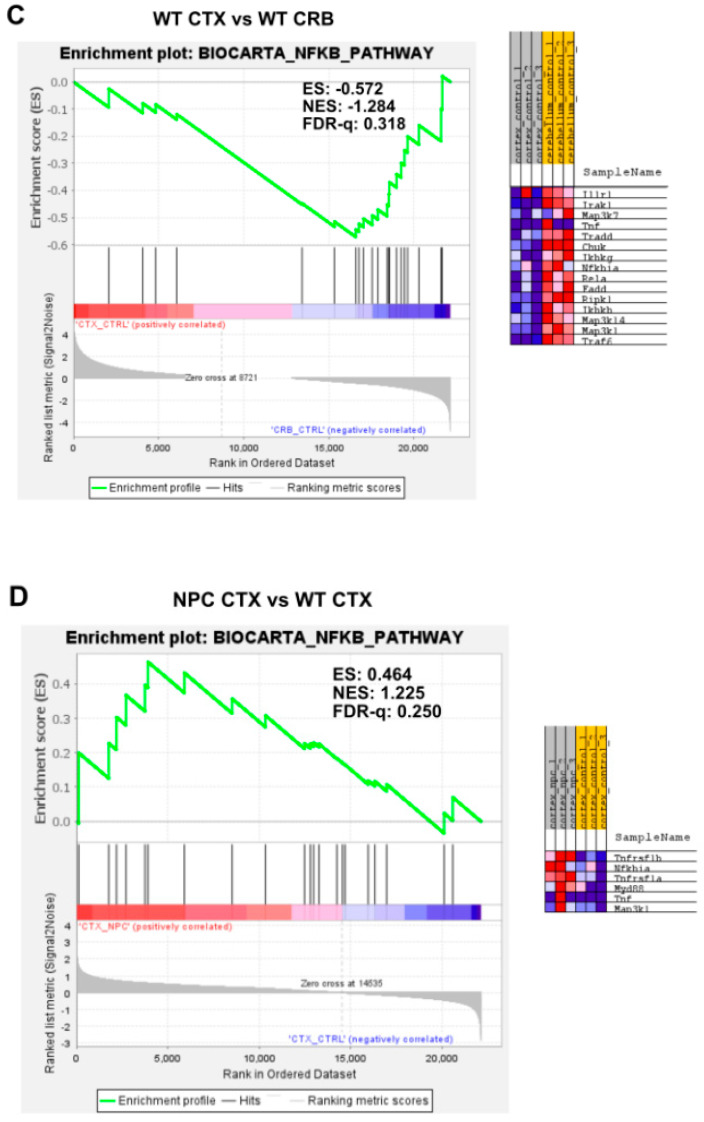
(**A**) Expanded version of Figure 3A from the main text with additional heatmap of core enriched genes. (**B**) Expanded version of Figure 3B from the main text with additional heatmap of core enriched genes. (**C**) Expanded version of Figure 3C from the main text with additional heatmap of core enriched gene. (**D**) Expanded version of Figure 3D from the main text with additional heatmap of core enriched gene.

**Figure A4:**
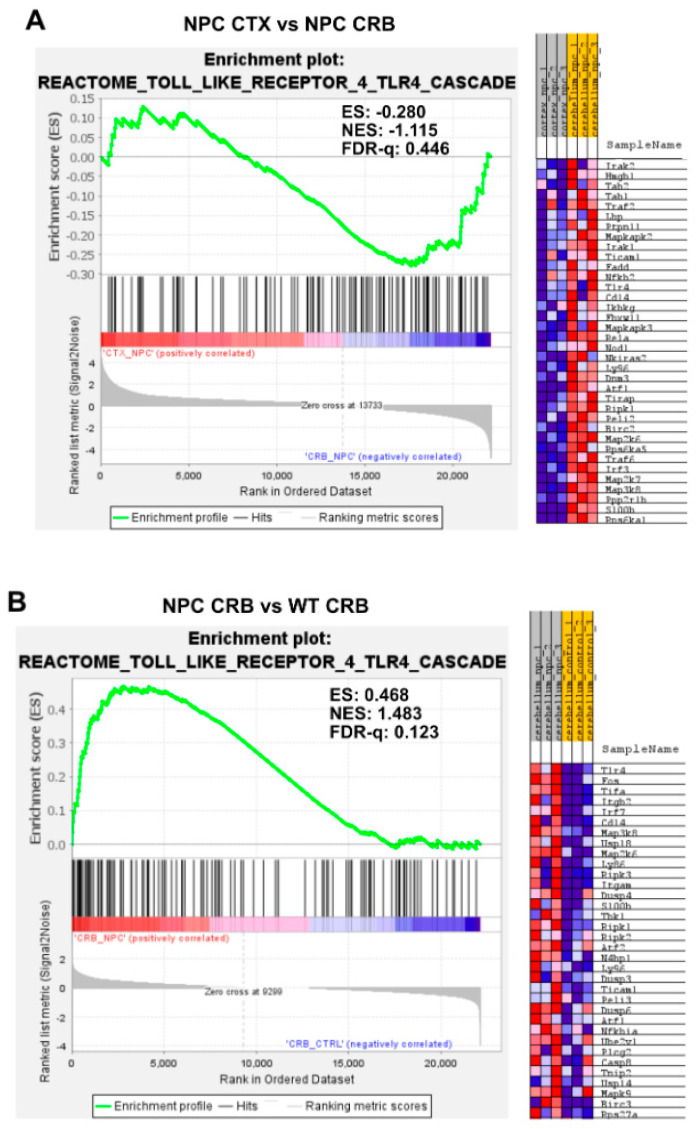

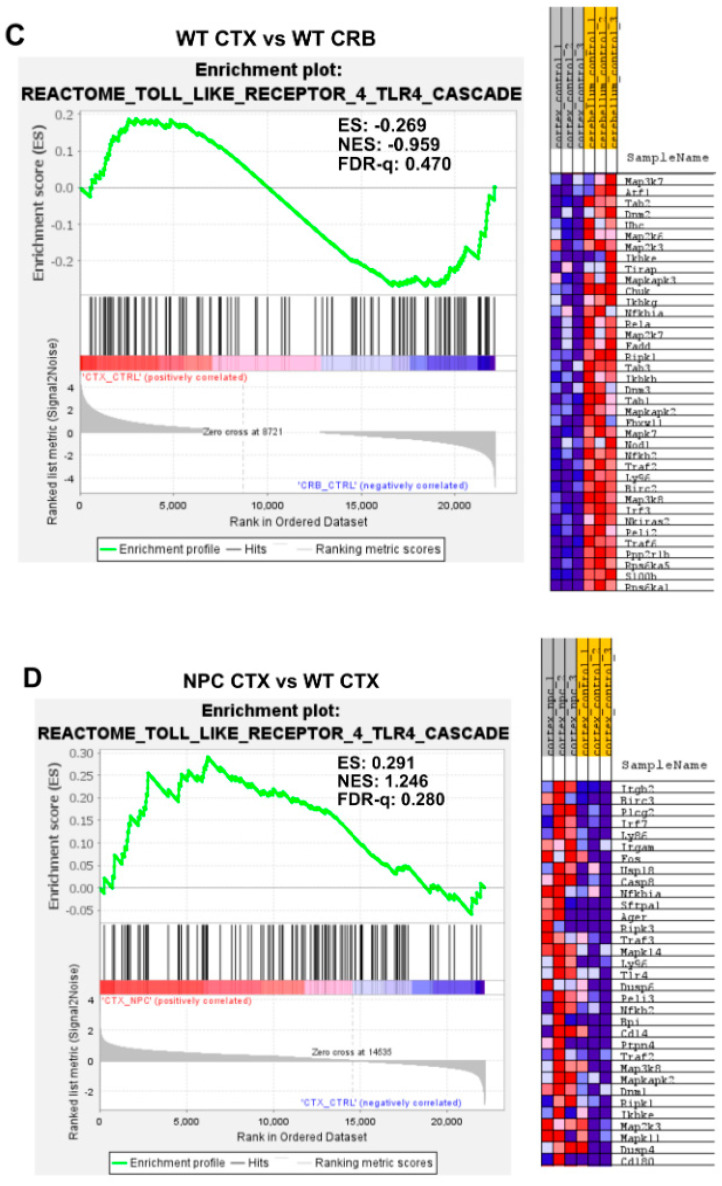
(**A**) Expanded version of Figure 4A from the main text with additional heatmap of core enriched genes. (**B**) Expanded version of Figure 4B from the main text with additional heatmap of core enriched genes. (**C**) Expanded version of Figure 4C from the main text with additional heatmap of core enriched genes. (**D**) Expanded version of Figure 4D from the main text with additional heatmap of core enriched genes.

**Figure A5:**
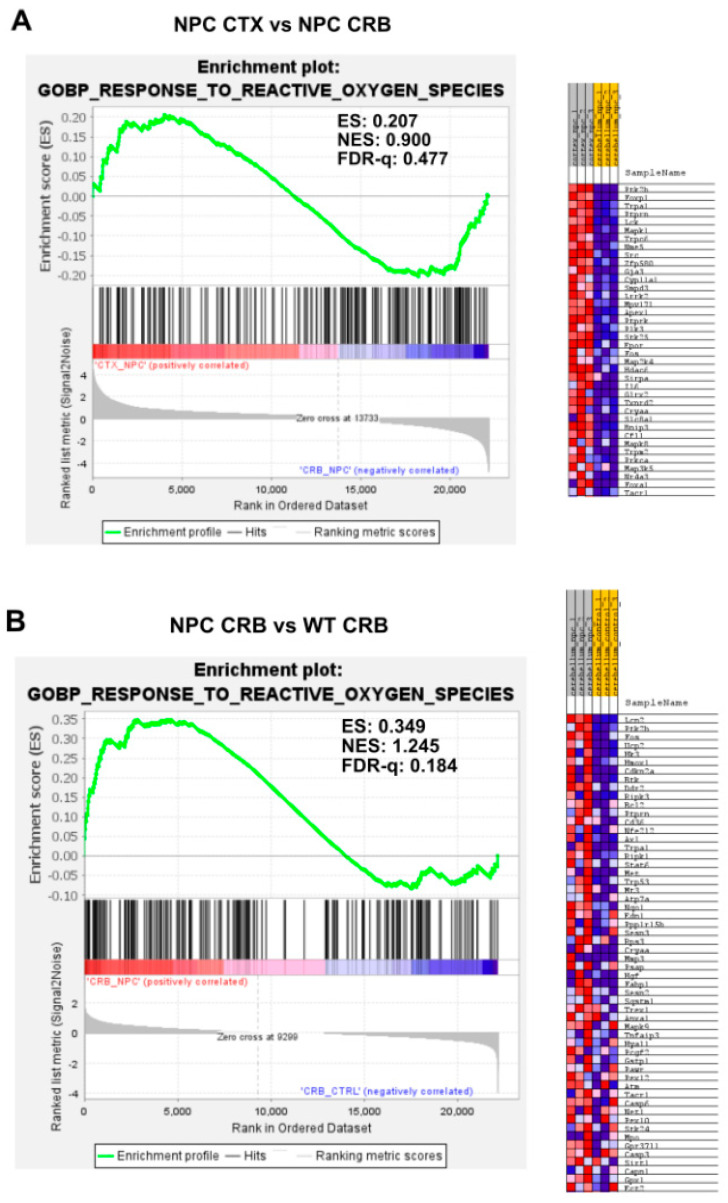

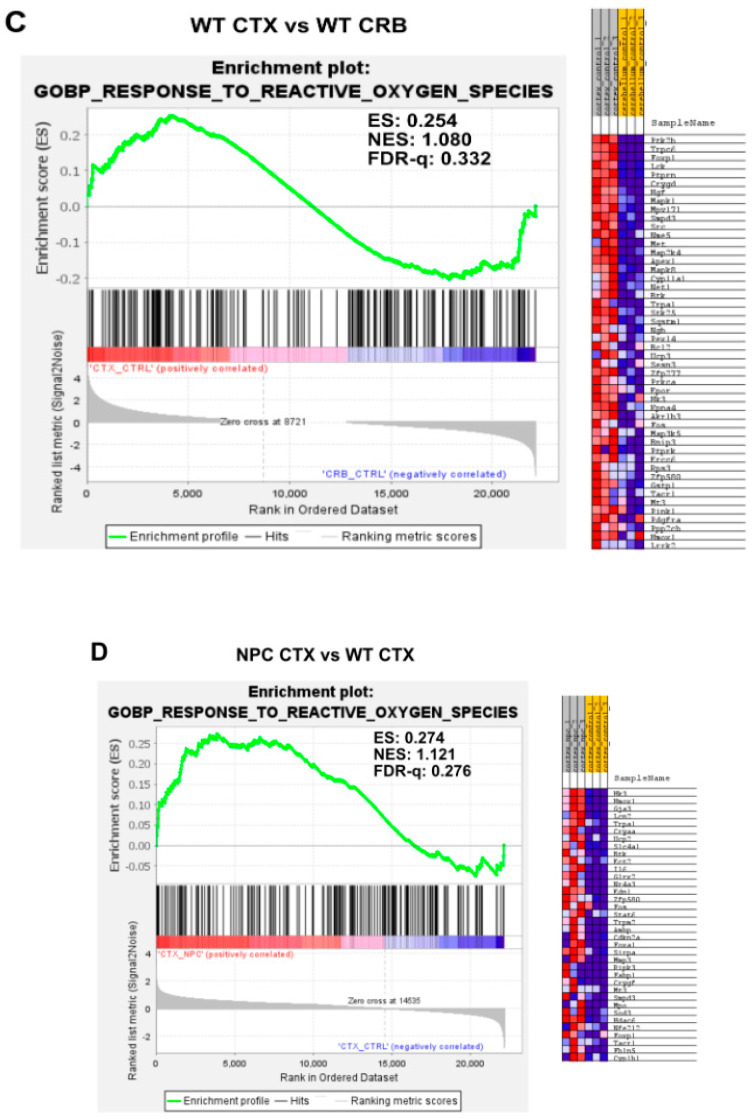
(**A**) Expanded version of Figure 5A from the main text with additional heatmap of core enriched genes. (**B**) Expanded version of Figure 5B from the main text with additional heatmap of core enriched gene. (**C**) Expanded version of Figure 5C from the main text with additional heatmap of core enriched gene. (**D**) Expanded version of Figure 5D from the main text with additional heatmap of core enriched genes.

**Figure A6:**
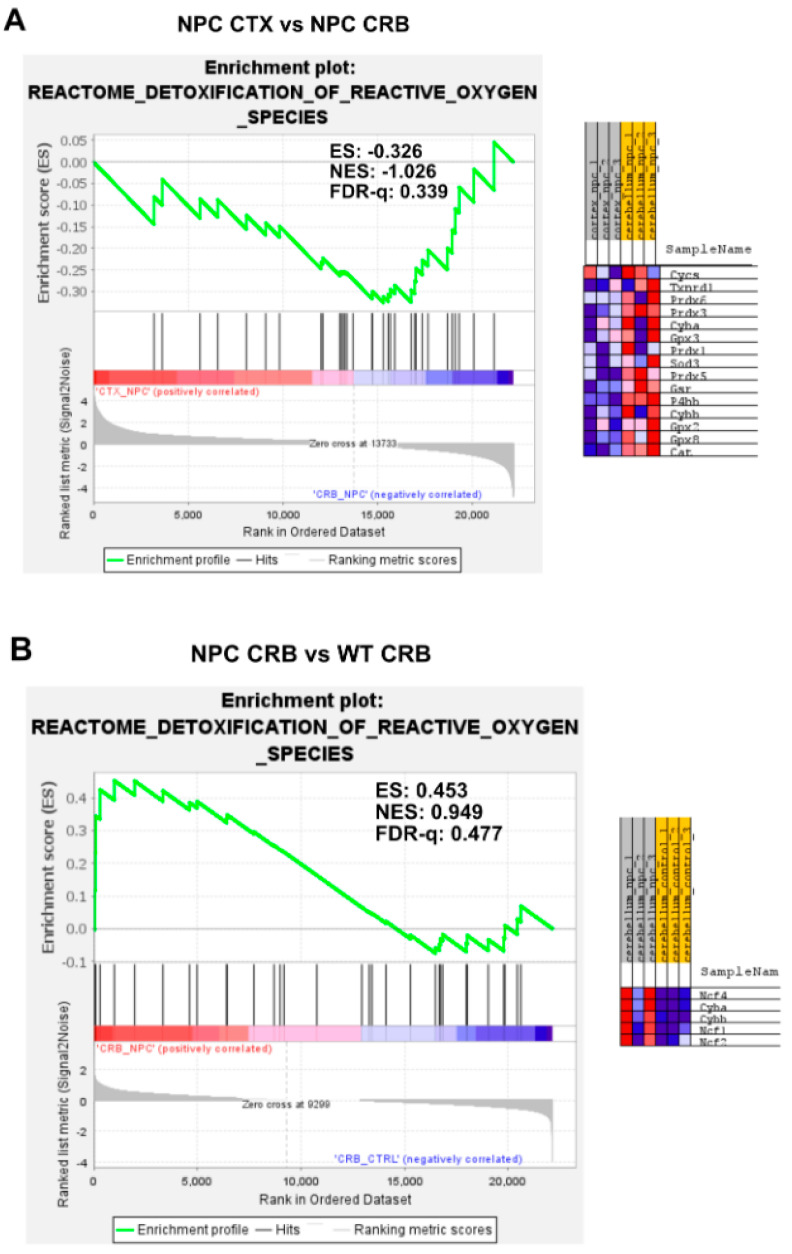

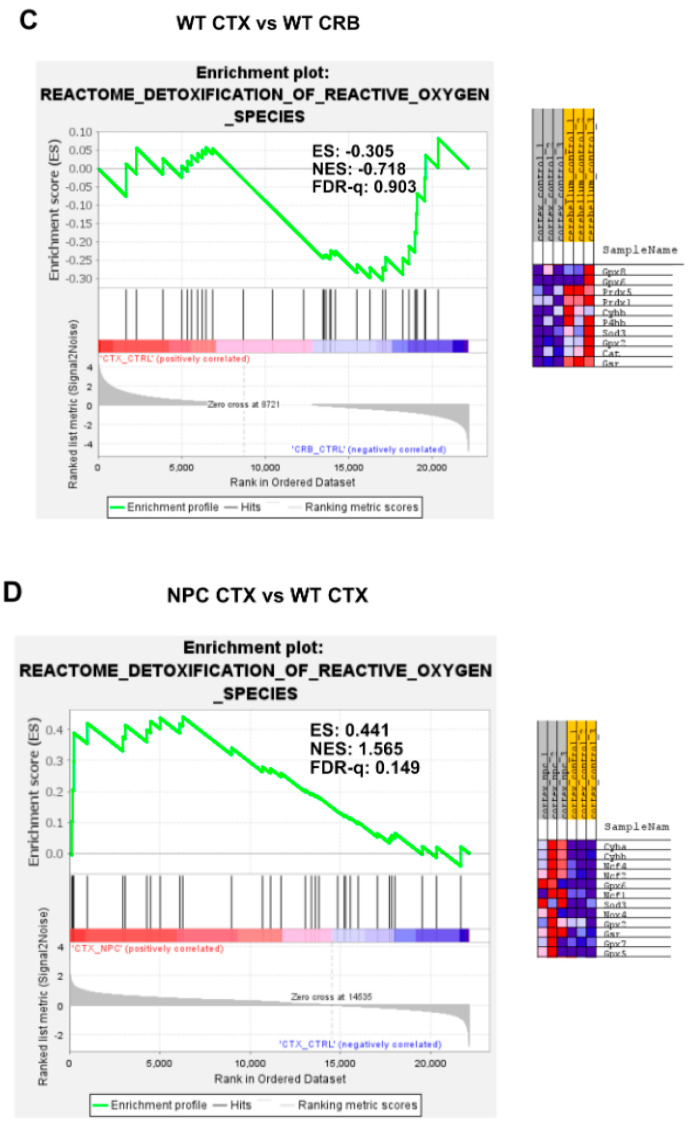
(**A**) Expanded version of Figure 6A from the main text with additional heatmap of core enriched genes. (**B**) Expanded version of Figure 6B from the main text with additional heatmap of core enriched genes. (**C**) Expanded version of Figure 6C from the main text with additional heatmap of core enriched genes. (**D**) Expanded version of Figure 6D from the main text with additional heatmap of core enriched genes.

**Figure A7:**
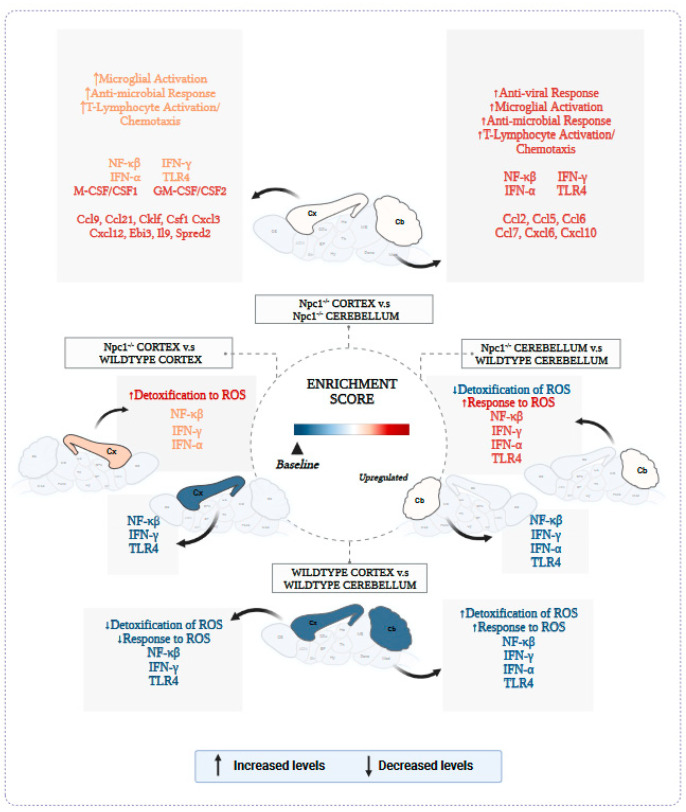
Expanded version of Figure 7 from the main text.

## Data Availability

The raw datasets that were used for the work presented in this article are not readily available because they are part of an ongoing study. Requests to access the datasets should be directed to S.S.

## Supplementary Materials

The following supporting information can be downloaded at https://www.mdpi.com/article/10.3390/genes15010101/s1, Supplementary Figure S1. IFN-γ- and IFN-α-responsive DEGs identified in the *Npc1^−/−^* cortical transcriptome; Supplementary Figure S2. Disease and Function Analysis of the *Npc1^−/−^* cortical transcriptome; Supplementary Table S1. DEG between wildtype and *Npc1^−/−^* cerebral cortex; Supplementary Table S2. Hallmark database gene sets; Supplementary Table S3. Differential expression of cytokine transcripts in the cerebral cortex vs. the cerebellum in the *Npc1^−/−^* mice.
