# Supplementary material for: Differential Interferon Signaling Regulation and Oxidative Stress Responses in the Cerebral Cortex and Cerebellum Could Account for the Spatiotemporal Pattern of Neurodegeneration in Niemann–Pick Disease Type C
Source: Genes (Basel). 2024 Jan 15;15(1):101. doi: 10.3390/genes15010101 (PMC10815326; doi:10.3390/genes15010101)
Supplement: Supplementary file 1 [file genes-15-00101-s001.zip › genes-2804419-supplementary.pdf]

SUPPLEMENTARY MATERIAL TOLAN ET AL

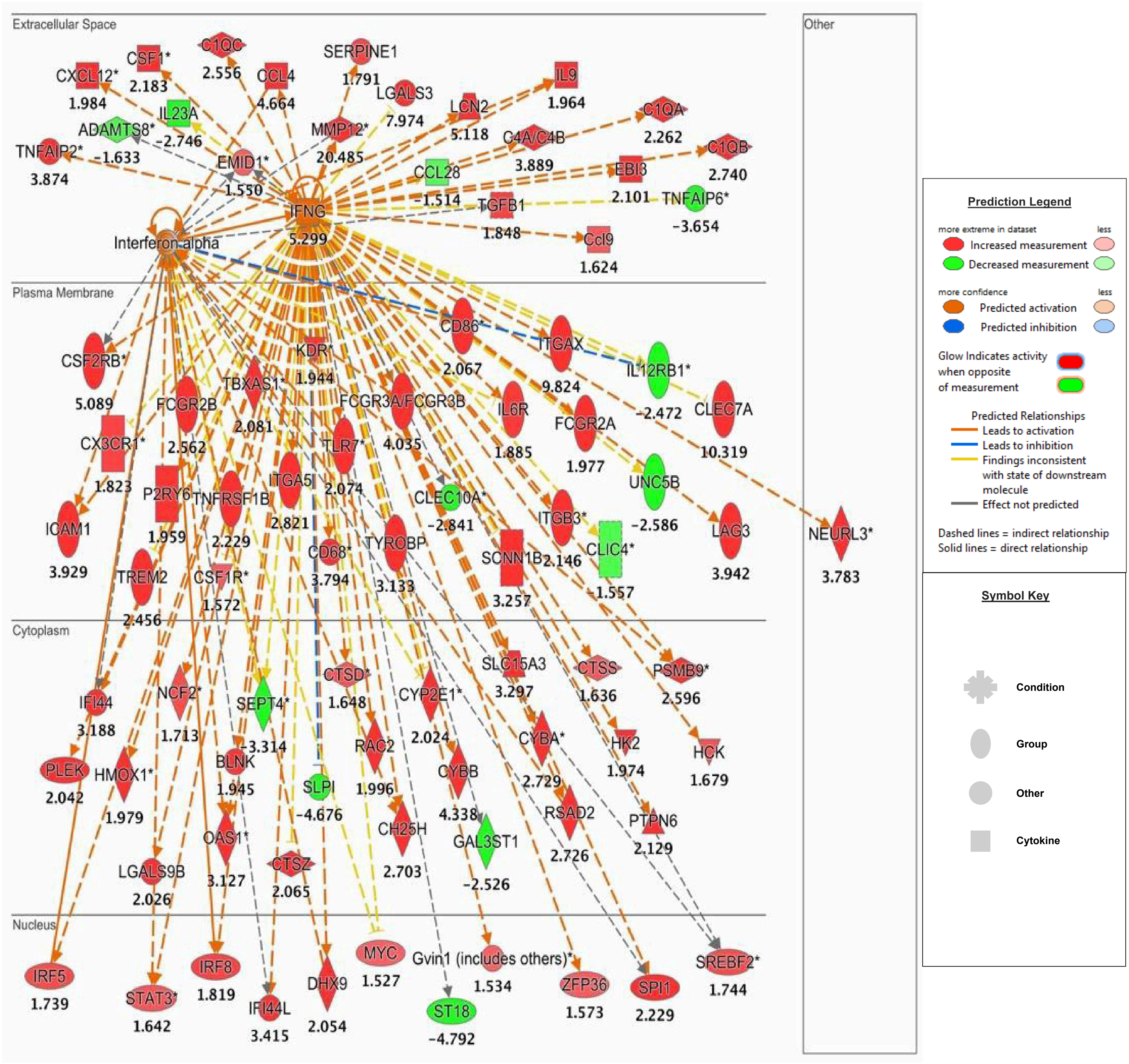

Supplementary Figure S1. IFN-γ- and IFN-α-responsive DEGs identified in the *Npc1*$^{-/-}$ cortical transcriptome. All differentially expressed genes (DEGs) are localized to their subcellular location. All plotted DEGs meet the significance cutoff of fold-change (ab-solute FC > 1.5) and p-value ($p < 0.05$).

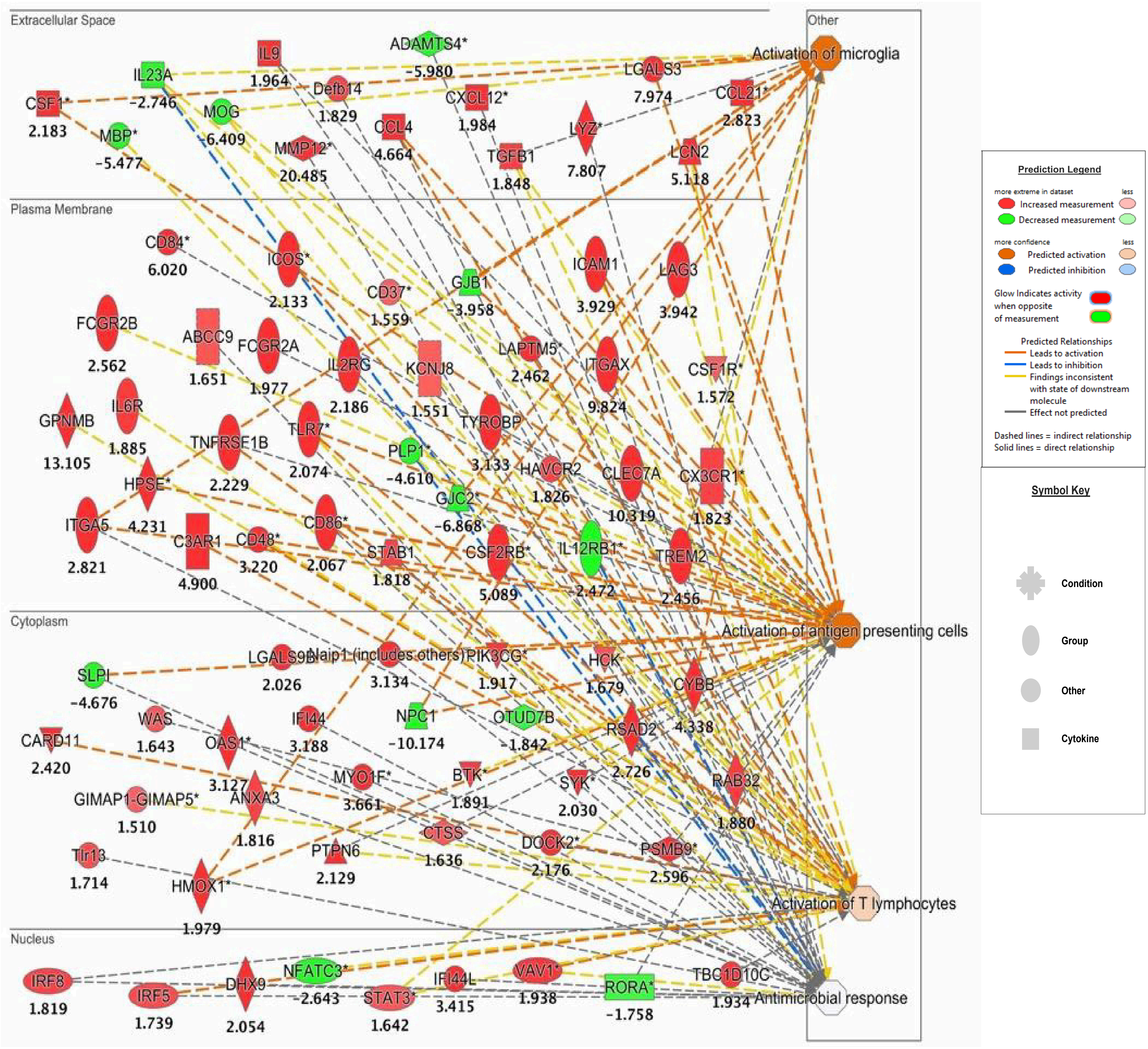

Supplementary figure S2. Disease and Function Analysis of the *Npc1*$^{-/-}$ cortical transcriptome. All differentially expressedgenes(DEGs) are localized to their sub-cellular location. All plotted DEGs meet the significance cutoff of fold-change (absolute FC > 1.5) and p-value ($p < 0.05$). A detailed key for IPA molecular shape, color, and interaction is provided in figure 2.

Supplementary Figure S3
Pandas Python package code used for data pre-processing, as described in Material and Methods section 2.3.

```
import pandas as pd
import numpy as np
import scipy.stats as stats
import matplotlib.pyplot as plt

# First we gather the data
# TODO: change this to where you have the datasets
ctx_df = pd.read_csv('/content/NPC_APP_Analysis_cortex.csv')
crb_df = pd.read_csv('/content/NPC_APP_Analysis.csv')

# Merge the cortex and cerebellum DataFrames
# by matching IDs
merged_df = pd.merge(
    crb_df,
    ctx_df,
    how="inner",
    on="Systematic",
    sort=False)

# Create a pruned DataFrame with only the columns we need
df = merged_df[['GeneSymbol', 'cerebellum_control_1',
'cerebellum_control_2',
               'cerebellum_control_3', 'cerebellum_npc_1',
'cerebellum_npc_2',
               'cerebellum_npc_3', 'cortex_control_1',
'cortex_control_2',
               'cortex_control_3', 'cortex_npc_1', 'cortex_npc_2',
               'cortex_npc_3']]

# Remove nill values
df.dropna()

# Create arrays to hold each group's columns
crb_ctrl = df[['cerebellum_control_1', 'cerebellum_control_2',
       'cerebellum_control_3']]

crb_npc = df[['cerebellum_npc_1', 'cerebellum_npc_2',
       'cerebellum_npc_3']]

ctx_ctrl = df[['cortex_control_1', 'cortex_control_2',
               'cortex_control_3']]

ctx_npc = df[['cortex_npc_1', 'cortex_npc_2',
               'cortex_npc_3']]
```

```
# Run an ANOVA to see which genes have significantly
# differential expression between groups.
# This is an extra step for verification as we run
# paired T-tests between the matchups of interest later on
df['F'], df['pval'] = stats.kruskal(crb_ctrl, crb_npc, ctx_ctrl,
ctx_npc, axis=1)

# Export the data
sig_df = df.loc[df['pval'] < 0.05]
sig_df.to_csv('/content/sig_df.csv', sep=',')

# Find mean of each group
df['crb_ctrl_mean'] = crb_ctrl.mean(axis=1)
df['crb_npc_mean'] = crb_npc.mean(axis=1)
df['ctx_ctrl_mean'] = ctx_ctrl.mean(axis=1)
df['ctx_npc_mean'] = ctx_npc.mean(axis=1)

# Find standard deviation of each group
df['crb_npc_std'] = crb_npc.std(axis=1)
df['crb_ctrl_std'] = crb_ctrl.std(axis=1)
df['ctx_npc_std'] = ctx_npc.std(axis=1)
df['ctx_ctrl_std'] = ctx_ctrl.std(axis=1)

# Do all the T-tests and move to a new column
# We are running these across columns for each row, hence axis=1
# and we do not assume equal variances, choosing to run
# Welsh's T-test instead of Student's
T_npc_ctx_vs_npc_crb =  stats.ttest_ind(ctx_npc, crb_npc, axis=1,
equal_var=False)
T_npc_crb_vs_ctrl_crb =  stats.ttest_ind(crb_npc, crb_ctrl, axis=1,
equal_var=False)
T_npc_ctx_vs_ctrl_crb =  stats.ttest_ind(ctx_npc, crb_ctrl, axis=1,
equal_var=False)
T_npc_ctx_vs_ctrl_ctx =  stats.ttest_ind(ctx_npc, ctx_ctrl, axis=1,
equal_var=False)

# Now convert them to columns in a dataframe
# stats.ttest_ind returns an array, with [1] being the p-value
a = pd.DataFrame({'T_npc_ctx_vs_npc_crb': T_npc_ctx_vs_npc_crb[1]})
b = pd.DataFrame({'T_npc_crb_vs_ctrl_crb': T_npc_crb_vs_ctrl_crb[1]})
c = pd.DataFrame({'T_npc_ctx_vs_ctrl_crb': T_npc_ctx_vs_ctrl_crb[1]})
d = pd.DataFrame({'T_npc_ctx_vs_ctrl_ctx': T_npc_ctx_vs_ctrl_ctx[1]})

df['npc_ctx_vs_npc_crb_pval'] = a
df['npc_crb_vs_ctrl_crb_pval'] = b
df['npc_ctx_vs_ctrl_crb_pval'] = c
df['npc_ctx_vs_ctrl_ctx_pval'] = d

# Now we prepare to run our DGE
# We are using Log2FC
```

```
groups = [df['crb_ctrl_mean'], df['ctx_ctrl_mean'],
          df['crb_npc_mean'], df['ctx_npc_mean']]

# NPC CTX vs NPC CRB
df['npc_ctx_npc_crb'] = np.log2(groups[3]/groups[2])

# NPC CTX vs CTRL CRB
df['npc_ctx_ctrl_crb'] = np.log2(groups[3]/groups[0])

# NPC CTX vs CTRL CTX
df['npc_ctx_ctrl_ctx'] = np.log2(groups[3]/groups[1])

# NPC CTX vs CTRL CTX
df['npc_crb_ctrl_crb'] = np.log2(groups[2]/groups[0])

# Finally, save our file as a CSV and use for further analysis
df.to_csv("/content/final_df.csv", sep=',')
```

Supplementary table S1 - Differentially expressed genes NPC1-/- cortex versus NPC1-/- cerebellum

Expr p-value	Expr Log Ratio	ID	Symbol	Entrez Gene Name	Location	Type(s)	Drug(s)
4.47E-02	-10.304	Gabra6	GABRA6	gamma-aminobutyric acid type A receptor subunit alpha6	Plasma Membrane	ion channel	flunitrazepam, sevoflurane, butobarbital, atropine/hyoscyamine/phenobarbital/scopolamine, phenobarbital, barbital, acetaminophen/butalbital/caffeine/codeine, hyoscyamine/phenobarbital, SEP 174559, desflurane, mephoba
8.3E-04	-10.046	Crtam	CRTAM	cytotoxic and regulatory T cell molecule	Plasma Membrane	other	
9.22E-03	-7.837	Ccnt2	CCNT2	cyclin T2	Nucleus	transcription regulator	
6.97E-05	-5.325	St18	ST18	ST18 C2H2C-type zinc finger transcription factor	Nucleus	transcription regulator	
2.05E-02	-4.806	Il16	IL16	interleukin 16	Extracellular Space	cytokine	
4.95E-02	-4.75	Eps8l2	EPS8L2	EPS8 like 2	Cytoplasm	other	
4.54E-02	-4.709	Tff3	TFF3	trefoil factor 3	Extracellular Space	other	
1.33E-02	-4.415	Kcng4	KCNG4	potassium voltage-gated channel modifier subfamily G member 4	Plasma Membrane	ion channel	
4.89E-03	-4.106	Gnmt	GNMT	glycine N-methyltransferase	Cytoplasm	enzyme	
5.86E-03	-3.901	Slc5a1	SLC5A1	solute carrier family 5 member 1	Plasma Membrane	transporter	YG1699, sotagliflozin
2.17E-03	-3.744	Svep1	SVEP1	sushi, von Willebrand factor type A, EGF and pentraxin domain containing 1	Cytoplasm	other	
5.72E-03	-3.706	Arg1	ARG1	arginase 1	Cytoplasm	enzyme	
3.37E-03	-3.657	Cnksr3	CNKSR3	CNKSR family member 3	Plasma Membrane	kinase	
2.99E-02	-3.592	Gchfr	GCHFR	GTP cyclohydrolase I feedback regulator	Cytoplasm	other	
5.33E-03	-3.494	Lhx5	LHX5	LIM homeobox 5	Nucleus	transcription regulator	
1.22E-02	-3.463	Slc1a6	SLC1A6	solute carrier family 1 member 6	Plasma Membrane	transporter	riluzole
3.27E-02	-3.457	Plekhf1	PLEKHF1	pleckstrin homology and FYVE domain containing 1	Cytoplasm	other	
5.67E-03	-3.408	Ccdc69	Ccdc69	coiled-coil domain containing 69	Other	other	
2.96E-02	-3.39	St14	ST14	ST14 transmembrane serine protease matriptase	Plasma Membrane	peptidase	
2.72E-04	-3.386	Serpinb1b	Serpinb1b	serine (or cysteine) peptidase inhibitor, clade B, member 1b	Extracellular Space	enzyme	
4.91E-02	-3.333	Ebf3	EBF3	EBF transcription factor 3	Nucleus	transcription regulator	
3.92E-03	-3.291	Tlx3	TLX3	T cell leukemia homeobox 3	Nucleus	transcription regulator	
2.23E-02	-3.201	Bmp5	BMP5	bone morphogenetic protein 5	Extracellular Space	growth factor	
5.26E-05	-3.153	Nrk	NRK	Nik related kinase	Other	kinase	
7.58E-04	-3.093	Slc22a4	SLC22A4	solute carrier family 22 member 4	Plasma Membrane	transporter	
3.91E-02	-3.092	Pax3	PAX3	paired box 3	Nucleus	transcription regulator	
2.46E-03	-3.044	Spag5	SPAG5	sperm associated antigen 5	Nucleus	peptidase	
2.01E-02	-3.026	Dusp10	DUSP10	dual specificity phosphatase 10	Nucleus	phosphatase	
5.89E-03	-2.867	Mapk13	MAPK13	mitogen-activated protein kinase 13	Cytoplasm	kinase	talmapimod
1.33E-02	-2.861	Slc2a4	SLC2A4	solute carrier family 2 member 4	Plasma Membrane	transporter	canakinumab/metformin/sulfonylurea, insulin glargine/lixisenatide/metformin, metformin/vildagliptin, metformin/rosiglitazone/sulfonylurea, metformin/sulfonylurea/vildagliptin, metformin/pioglitazone/sulfonylurea, INS/metform
4.59E-02	-2.823	Fzd7	FZD7	frizzled class receptor 7	Plasma Membrane	G-protein coupled receptor	vantictumab
3.78E-03	-2.779	Marveld2	MARVELD2	MARVEL domain containing 2	Plasma Membrane	other	
7.83E-03	-2.761	Scx	SCX	scleraxis bHLH transcription factor	Nucleus	transcription regulator	
4.31E-03	-2.659	Opalin	OPALIN	oligodendrocytic myelin paranodal and inner loop protein	Cytoplasm	other	
3.87E-03	-2.659	Sp5	SP5	Sp5 transcription factor	Nucleus	transcription regulator	
4.24E-02	-2.644	Comp	COMP	cartilage oligomeric matrix protein	Extracellular Space	other	
1.67E-02	-2.64	Exph5	EXPH5	exophilin 5	Cytoplasm	other	
3.99E-02	-2.623	Itgb7	ITGB7	integrin subunit beta 7	Plasma Membrane	transmembrane receptor	vedolizumab
2.24E-02	-2.608	Sdc1	SDC1	syndecan 1	Plasma Membrane	enzyme	indatuximab ravtansine
3.92E-02	-2.604	Lingo4	LINGO4	leucine rich repeat and Ig domain containing 4	Other	other	
1.12E-03	-2.602	Ldlrap1	LDLRAP1	low density lipoprotein receptor adaptor protein 1	Cytoplasm	transporter	
3E-03	-2.592	Ebf2	EBF2	EBF transcription factor 2	Nucleus	transcription regulator	
1.91E-03	-2.565	6430571L13Rik	C3orf18	chromosome 3 open reading frame 18	Other	other	
1.75E-02	-2.506	Car8	CA8	carbonic anhydrase 8	Cytoplasm	enzyme	
4.87E-02	-2.458	Utp6	UTP6	UTP6 small subunit processome component	Nucleus	other	
2.49E-02	-2.426	Slc41a3	SLC41A3	solute carrier family 41 member 3	Plasma Membrane	transporter	
1.17E-02	-2.402	Car14	CA14	carbonic anhydrase 14	Plasma Membrane	enzyme	hydrochlorothiazide, trichloromethiazide, sulfacetamide, benzthiazide, topiramate, chlorothiazide, chlorthalidone, acetazolamide, methazolamide
4.3E-02	-2.361	Tmem88b	TMEM88B	transmembrane protein 88B	Other	other	
1.29E-02	-2.346	Esrrb	ESRRB	estrogen related receptor beta	Nucleus	transcription regulator	
2.17E-02	-2.344	Hsd11b1	HSD11B1	hydroxysteroid 11-beta dehydrogenase 1	Cytoplasm	enzyme	
4.67E-02	-2.308	S1pr2	S1PR2	sphingosine-1-phosphate receptor 2	Plasma Membrane	G-protein coupled receptor	
5.37E-03	-2.307	Kat2b	KAT2B	lysine acetyltransferase 2B	Nucleus	enzyme	
6.3E-03	-2.285	Tspan2	TSPAN2	tetraspanin 2	Extracellular Space	other	
5.64E-03	-2.266	Ucma	UCMA	upper zone of growth plate and cartilage matrix associated	Extracellular Space	other	
2.45E-02	-2.264	Spata20	SPATA20	spermatogenesis associated 20	Other	other	
5.51E-03	-2.257	Atoh8	ATOH8	atonal bHLH transcription factor 8	Nucleus	transcription regulator	
5.68E-03	-2.193	Cyp27a1	CYP27A1	cytochrome P450 family 27 subfamily A member 1	Cytoplasm	enzyme	
3.26E-02	-2.19	Nkapl	NKAPL	NFKB activating protein like	Other	other	
6.93E-03	-2.184	Pnpla3	PNPLA3	patatin like phospholipase domain containing 3	Cytoplasm	enzyme	
2.4E-02	-2.163	Rassf6	RASSF6	Ras association domain family member 6	Other	other	
3.91E-02	-2.156	Trpc3	TRPC3	transient receptor potential cation channel subfamily C member 3	Plasma Membrane	ion channel	
3.26E-03	-2.123	Metrn	METRN	meteorin, glial cell differentiation regulator	Extracellular Space	other	
6.34E-04	-2.116	Kcnk3	KCNK3	potassium two pore domain channel subfamily K member 3	Plasma Membrane	ion channel	doxapram, amiodarone, halothane, nicorandil
3.27E-02	-2.101	Bub1	BUB1	BUB1 mitotic checkpoint serine/threonine kinase	Nucleus	kinase	
3.27E-02	-2.072	Smarcd2	SMARCD2	SWI/SNF related, matrix associated, actin dependent regulator of chromatin,	Nucleus	transcription regulator	
9.98E-03	-2.035	Cldn14	CLDN14	claudin 14	Plasma Membrane	other	
6.35E-03	-2.009	Sct	Sct	secretin	Extracellular Space	other	
1.89E-02	-1.985	Rbpjl	RBPJL	recombination signal binding protein for immunoglobulin kappa J region like	Nucleus	transcription regulator	
4.75E-03	-1.974	Zfpm2	ZFPM2	zinc finger protein, FOG family member 2	Nucleus	transcription regulator	
3.23E-04	-1.971	Cox6b2	COX6B2	cytochrome c oxidase subunit 6B2	Cytoplasm	enzyme	
3.15E-03	-1.971	B3gnt8	B3GNT8	UDP-GlcNAc:betaGal beta-1,3-N-acetylglucosaminyltransferase 8	Cytoplasm	enzyme	
7.5E-04	-1.966	Slc34a3	SLC34A3	solute carrier family 34 member 3	Plasma Membrane	transporter	
2.6E-02	-1.942	Syt9	SYT9	synaptotagmin 9	Plasma Membrane	transporter	
2.49E-03	-1.941	Kif26b	KIF26B	kinesin family member 26B	Other	other	
7.26E-03	-1.939	Rffl	RFFL	ring finger and FYVE like domain containing E3 ubiquitin protein ligase	Cytoplasm	enzyme	
4.99E-02	-1.938	Fzd1	FZD1	frizzled class receptor 1	Plasma Membrane	G-protein coupled receptor	vantictumab
4.84E-02	-1.927	Cpm	CPM	carboxypeptidase M	Plasma Membrane	peptidase	
9.69E-03	-1.924	Dhx40	DHX40	DEAH-box helicase 40	Other	enzyme	
1.68E-02	-1.918	Sned1	SNED1	sushi, nidogen and EGF like domains 1	Plasma Membrane	other	
1.6E-02	-1.895	Slc6a12	SLC6A12	solute carrier family 6 member 12	Plasma Membrane	transporter	tiagabine
1.22E-02	-1.874	Ryr1	RYR1	ryanodine receptor 1	Cytoplasm	ion channel	dantrolene
1.59E-02	-1.856	Traf3ip2	TRAF3IP2	TRAF3 interacting protein 2	Cytoplasm	enzyme	
2.7E-02	-1.834	Aard	AARD	alanine and arginine rich domain containing protein	Other	other	
4.71E-03	-1.834	Mamdc2	MAMDC2	MAM domain containing 2	Extracellular Space	other	
3.27E-02	-1.828	Ms4a6c	Ms4a6c	membrane-spanning 4-domains, subfamily A, member 6C	Cytoplasm	other	
3.23E-02	-1.828	Cst7	CST7	cystatin F	Extracellular Space	other	
6.97E-04	-1.822	Zfp536	ZNF536	zinc finger protein 536	Nucleus	transcription regulator	
3.37E-03	-1.814	Cda	CDA	cytidine deaminase	Nucleus	enzyme	cedazuridine/decitabine, cytidine deaminase inhibitor, 5-azacytidine/cedazuridine, cedazuridine
6.41E-03	-1.813	Rbp7	RBP7	retinol binding protein 7	Cytoplasm	other	
2.3E-02	-1.803	Extl2	EXTL2	exostosin like glycosyltransferase 2	Cytoplasm	enzyme	
2.26E-03	-1.766	A230072C01Rik	A230072C01	RIKEN cDNA A230072C01 gene	Other	other	
2.58E-02	-1.766	Clec7a	CLEC7A	C-type lectin domain containing 7A	Plasma Membrane	transmembrane receptor	
3.9E-03	-1.765	Cercam	CERCAM	cerebral endothelial cell adhesion molecule	Other	other	
5.54E-03	-1.762	Hk2	HK2	hexokinase 2	Cytoplasm	kinase	
6.4E-03	-1.757	Sema4g	SEMA4G	semaphorin 4G	Plasma Membrane	other	
1.38E-02	-1.745	Dhx58	DHX58	DExH-box helicase 58	Cytoplasm	enzyme	
1.46E-02	-1.737	Mycbp	MYCBP	MYC binding protein	Nucleus	transcription regulator	
4.26E-02	-1.699	Cdr2	CDR2	cerebellar degeneration related protein 2	Cytoplasm	other	
3.1E-04	-1.698	Bik	BIK	BCL2 interacting killer	Cytoplasm	other	
1.37E-03	-1.68	Hdc	HDC	histidine decarboxylase	Cytoplasm	enzyme	
2.32E-02	-1.676	Kctd8	KCTD8	potassium channel tetramerization domain containing 8	Other	other	
2.69E-02	-1.652	Zfp773	Zfp773	zinc finger protein 773	Other	other	
2.73E-03	-1.652	Slc27a5	SLC27A5	solute carrier family 27 member 5	Cytoplasm	transporter	
1.89E-02	-1.65	Npb	NPB	neuropeptide B	Extracellular Space	other	
1.06E-02	-1.647	Vim	VIM	vimentin	Cytoplasm	other	pritumumab
8.96E-03	-1.645	Pdlim3	Pdlim3	PDZ and LIM domain 3	Plasma Membrane	other	
1.42E-02	-1.624	Ceacam10	Ceacam10	CEA cell adhesion molecule 10	Extracellular Space	other	
3.92E-03	-1.609	Pcf11	PCF11	PCF11 cleavage and polyadenylation factor subunit	Nucleus	other	
1.07E-02	-1.604	Gm833	Gm833	predicted gene 833	Other	other	
3.05E-02	-1.6	Cd5	CD5	CD5 molecule	Plasma Membrane	transmembrane receptor	
4.8E-02	-1.594	Snrk	SNRK	SNF related kinase	Cytoplasm	kinase	
5.17E-03	-1.589	Dact1	DACT1	dishevelled binding antagonist of beta catenin 1	Cytoplasm	other	
1.45E-02	-1.587	Slc14a1	SLC14A1	solute carrier family 14 member 1 (Kidd blood group)	Plasma Membrane	transporter	
2.16E-02	-1.585	Tmtc2	TMTC2	transmembrane O-mannosyltransferase targeting cadherins 2	Cytoplasm	enzyme	
3.99E-03	-1.584	Plek2	PLEK2	pleckstrin 2	Plasma Membrane	other	
1.61E-02	-1.566	Itgax	ITGAX	integrin subunit alpha X	Plasma Membrane	transmembrane receptor	
1.95E-02	-1.561	Tnni3	TNNI3	troponin I3, cardiac type	Cytoplasm	transporter	
9.55E-03	-1.547	Arhgef10	ARHGEF10	Rho guanine nucleotide exchange factor 10	Cytoplasm	enzyme	
4.19E-03	-1.536	Cidea	CIDEA	cell death inducing DFFA like effector a	Cytoplasm	other	
5.99E-03	-1.536	Slc25a13	SLC25A13	solute carrier family 25 member 13	Cytoplasm	transporter	
1.97E-02	-1.518	Omd	OMD	osteomodulin	Extracellular Space	other	
2.72E-02	-1.503	Itih2	ITIH2	inter-alpha-trypsin inhibitor heavy chain 2	Extracellular Space	other	
6.33E-04	1.502	Senp5	SENP5	SUMO specific peptidase 5	Nucleus	peptidase	
8.5E-03	1.505	Rilpl1	RILPL1	Rab interacting lysosomal protein like 1	Cytoplasm	other	
3.42E-02	1.505	Spata21	SPATA21	spermatogenesis associated 21	Other	other	
8.27E-03	1.526	Slc1a1	SLC1A1	solute carrier family 1 member 1	Plasma Membrane	transporter	riluzole

9.84E-03	1.529	Lmcd1	LMCD1	LIM and cysteine rich domains 1	Cytoplasm	transcription regulator	
1.23E-02	1.55	D130043K22Rik	KIAA0319	KIAA0319	Plasma Membrane	other	
1.91E-02	1.552	Ntrk2	NTRK2	neurotrophic receptor tyrosine kinase 2	Plasma Membrane	kinase	ONO-7579, larotrectinib, AZ-23, NOV1601, taletrectinib, belizatinib, cabozantinib/nivolumab, LOXO-195, PBI-200, cabozantinib, bevacizumab/entrectinib, lorlatinib, entrectinib, PLX7486, repotrectinib, cabozantinib/tyrosine k
5.6E-03	1.556	Rtn4r	RTN4R	reticulon 4 receptor	Plasma Membrane	transmembrane receptor	
3.46E-02	1.556	Tmem30c	Tmem30c	transmembrane protein 30C	Other	other	
2.62E-02	1.564	Cmpk2	CMPK2	cytidine/uridine monophosphate kinase 2	Cytoplasm	kinase	
4.04E-03	1.564	Iqgap2	IQGAP2	IQ motif containing GTPase activating protein 2	Cytoplasm	other	
5.84E-03	1.567	Stat4	STAT4	signal transducer and activator of transcription 4	Nucleus	transcription regulator	
4.08E-02	1.572	Clvs2	CLVS2	clavesin 2	Cytoplasm	transporter	
3.45E-04	1.576	Nsg2	NSG2	neuronal vesicle trafficking associated 2	Cytoplasm	other	
3.92E-03	1.58	Smarcd1	SMARCD1	SWI/SNF related, matrix associated, actin dependent regulator of chromatin,	Nucleus	transcription regulator	
4.61E-02	1.586	Fhad1	FHAD1	forkhead associated phosphopeptide binding domain 1	Other	other	
4.62E-02	1.593	Osbpl10	OSBPL10	oxysterol binding protein like 10	Cytoplasm	transporter	
1.31E-02	1.594	Sult1e1	SULT1E1	sulfotransferase family 1E member 1	Cytoplasm	enzyme	estrogen sulfotransferase inhibitor
1.22E-04	1.608	Ltk	LTK	leukocyte receptor tyrosine kinase	Plasma Membrane	kinase	lorlatinib
1.53E-02	1.61	Dio1	DIO1	iodothyronine deiodinase 1	Cytoplasm	enzyme	propylthiouracil
1.73E-03	1.613	Svopl	SVOPL	SVOP like	Other	other	
5.66E-03	1.617	Slc36a3	SLC36A3	solute carrier family 36 member 3	Other	transporter	
3.63E-02	1.621	Kcnk2	KCNK2	potassium two pore domain channel subfamily K member 2	Plasma Membrane	ion channel	
2.48E-03	1.623	Scnn1a	SCNN1A	sodium channel epithelial 1 subunit alpha	Plasma Membrane	ion channel	hydrochlorothiazide/triamterene, amiloride, triamterene, amiloride/hydrochlorothiazide
1.33E-02	1.623	Ankrd35	ANKRD35	ankyrin repeat domain 35	Other	other	
4.96E-02	1.628	Ccr5	CCR5	C-C motif chemokine receptor 5	Plasma Membrane	G-protein coupled receptor	leronlimab, ancriviroc, MK-0812, vicriviroc, maraviroc, BMS-813160, nagrestipen
2.09E-02	1.631	Magi3	MAGI3	membrane associated guanylate kinase, WW and PDZ domain containing 3	Cytoplasm	kinase	
1.01E-02	1.643	Grm3	GRM3	glutamate metabotropic receptor 3	Plasma Membrane	G-protein coupled receptor	fasoracetam
2.15E-02	1.651	Ankrd27	ANKRD27	ankyrin repeat domain 27	Cytoplasm	other	
3.68E-02	1.654	Thsd7b	THSD7B	thrombospondin type 1 domain containing 7B	Other	other	
8.1E-03	1.66	Pbx4	PBX4	PBX homeobox 4	Nucleus	transcription regulator	
1.72E-02	1.677	B4galt2	B4GALT2	beta-1,4-galactosyltransferase 2	Cytoplasm	enzyme	
3.24E-02	1.678	Tshz3	TSHZ3	teashirt zinc finger homeobox 3	Nucleus	transcription regulator	
2.08E-02	1.684	Caly	CALY	calcyon neuron specific vesicular protein	Plasma Membrane	transmembrane receptor	trifluoperazine
2.81E-02	1.69	Arsj	ARSJ	arylsulfatase family member J	Extracellular Space	enzyme	
6.22E-03	1.707	Rpp25	RPP25	ribonuclease P and MRP subunit p25	Nucleus	enzyme	
5.52E-03	1.737	Fosl1	FOSL1	FOS like 1, AP-1 transcription factor subunit	Nucleus	transcription regulator	farletuzumab ecteribulin
1.83E-02	1.741	Wfs1	WFS1	wolframin ER transmembrane glycoprotein	Cytoplasm	enzyme	
3.58E-02	1.747	Kcnc2	KCNC2	potassium voltage-gated channel subfamily C member 2	Plasma Membrane	ion channel	dalfampridine
4.56E-03	1.771	Engase	ENGASE	endo-beta-N-acetylglucosaminidase	Cytoplasm	enzyme	
4.42E-02	1.776	Grik5	GRIK5	glutamate ionotropic receptor kainate type subunit 5	Plasma Membrane	ion channel	
3.67E-03	1.778	Amn	AMN	amnion associated transmembrane protein	Plasma Membrane	other	
1.94E-02	1.791	Muc20	MUC20	mucin 20, cell surface associated	Plasma Membrane	other	
1.43E-02	1.795	Sort1	SORT1	sortilin 1	Plasma Membrane	G-protein coupled receptor	TH1902
4.57E-02	1.802	Ppp1r1a	PPP1R1A	protein phosphatase 1 regulatory inhibitor subunit 1A	Cytoplasm	phosphatase	
4.4E-02	1.803	Acot4	ACOT4	acyl-CoA thioesterase 4	Cytoplasm	enzyme	
3.74E-02	1.814	Apob	APOB	apolipoprotein B	Extracellular Space	transporter	mipomersen
4.96E-02	1.831	Myh4	MYH4	myosin heavy chain 4	Cytoplasm	enzyme	
1.29E-02	1.853	Kcnb2	KCNB2	potassium voltage-gated channel subfamily B member 2	Plasma Membrane	ion channel	dalfampridine
8.65E-04	1.867	Klf14	KLF14	KLF transcription factor 14	Nucleus	transcription regulator	
2.82E-02	1.869	Spag6	Spag6	sperm associated antigen 6	Nucleus	other	
9.78E-03	1.871	Gucy2c	GUCY2C	guanylate cyclase 2C	Plasma Membrane	kinase	dolcanatide, linaclotide, indusatumab vedotin, plecanatide, PF-07062119
2.21E-02	1.872	Dleu7	DLEU7	deleted in lymphocytic leukemia 7	Other	other	
1.95E-02	1.899	Cryga	CRYGA	crystallin gamma A	Extracellular Space	other	
1.51E-02	1.912	Arnt2	ARNT2	aryl hydrocarbon receptor nuclear translocator 2	Nucleus	transcription regulator	
5.92E-03	1.914	Stard8	STARD8	StAR related lipid transfer domain containing 8	Cytoplasm	other	
5.47E-04	1.929	Lmtk2	LMTK2	lemur tyrosine kinase 2	Cytoplasm	kinase	
4.2E-02	1.931	Tac1	TAC1	tachykinin precursor 1	Extracellular Space	other	
1.95E-02	1.968	Npy5r	NPY5R	neuropeptide Y receptor Y5	Plasma Membrane	G-protein coupled receptor	
1.2E-02	1.968	Acta1	ACTA1	actin alpha 1, skeletal muscle	Cytoplasm	other	
1.57E-02	1.977	Shisa9	SHISA9	shisa family member 9	Plasma Membrane	other	
4.52E-02	1.98	Olfm2	OLFM2	olfactomedin 2	Cytoplasm	other	
9.2E-04	1.983	Nxph3	NXPH3	neurexophilin 3	Extracellular Space	other	
1.03E-02	1.983	Il17b	IL17B	interleukin 17B	Extracellular Space	cytokine	
4.03E-03	1.988	Plcz1	PLCZ1	phospholipase C zeta 1	Cytoplasm	enzyme	
1.77E-02	1.992	Pkp2	PKP2	plakophilin 2	Plasma Membrane	other	
2.91E-03	2.006	Smpd3	SMPD3	sphingomyelin phosphodiesterase 3	Cytoplasm	enzyme	
3.32E-03	2.02	Dmrtb1	DMRTB1	DMRT like family B with proline rich C-terminal 1	Nucleus	transcription regulator	
1.59E-02	2.021	Celsr1	CELSR1	cadherin EGF LAG seven-pass G-type receptor 1	Plasma Membrane	G-protein coupled receptor	
3.76E-02	2.026	Dclk3	DCLK3	doublecortin like kinase 3	Cytoplasm	kinase	
4.71E-02	2.053	Pcdh19	PCDH19	protocadherin 19	Extracellular Space	other	
3.99E-02	2.059	Ccdc65	CCDC65	coiled-coil domain containing 65	Cytoplasm	other	
4.81E-02	2.066	Necab2	NECAB2	N-terminal EF-hand calcium binding protein 2	Cytoplasm	other	
1.65E-03	2.086	Itgb6	ITGB6	integrin subunit beta 6	Plasma Membrane	other	
7.5E-03	2.089	Ppp1r3g	PPP1R3G	protein phosphatase 1 regulatory subunit 3G	Cytoplasm	other	
2.99E-02	2.091	Ccdc103	CCDC103	coiled-coil domain containing 103	Cytoplasm	other	
1.94E-02	2.119	Lypd1	LYPD1	LY6/PLAUR domain containing 1	Plasma Membrane	G-protein coupled receptor	
2.2E-03	2.137	Fras1	FRAS1	Fraser extracellular matrix complex subunit 1	Extracellular Space	other	
4.71E-03	2.137	Clec2h	Clec2e/Clec2	C-type lectin domain family 2, member e	Plasma Membrane	transmembrane receptor	
4.51E-03	2.149	2010204K13Rik	2010204K13	RIKEN cDNA 2010204K13 gene	Other	other	
1.79E-02	2.153	Dgat2	DGAT2	diacylglycerol O-acyltransferase 2	Cytoplasm	enzyme	
1.62E-02	2.156	Nrarp	NRARP	NOTCH regulated ankyrin repeat protein	Nucleus	transcription regulator	
4.85E-03	2.183	Pnmt	PNMT	phenylethanolamine N-methyltransferase	Cytoplasm	enzyme	
1.6E-02	2.186	Kcns1	KCNS1	potassium voltage-gated channel modifier subfamily S member 1	Plasma Membrane	ion channel	
3.54E-02	2.206	Cdh13	CDH13	cadherin 13	Plasma Membrane	other	
3.5E-02	2.216	Gm9767	Gm9767	predicted gene 9767	Other	other	
3.38E-02	2.216	Tmem30b	TMEM30B	transmembrane protein 30B	Cytoplasm	transporter	
1.36E-02	2.228	Ppm1e	PPM1E	protein phosphatase, Mg2+/Mn2+ dependent 1E	Nucleus	phosphatase	
2.97E-02	2.232	Hao2	HAO2	hydroxyacid oxidase 2	Cytoplasm	enzyme	
7.8E-03	2.235	Dlx6	DLX6	distal-less homeobox 6	Nucleus	transcription regulator	
1.15E-02	2.251	Crb2	CRB2	crumbs cell polarity complex component 2	Extracellular Space	other	
4.24E-03	2.323	Lrrc40	LRRC40	leucine rich repeat containing 40	Nucleus	other	
4.32E-02	2.324	AI593442	C11orf87	chromosome 11 open reading frame 87	Other	other	
1.38E-02	2.371	Tnfaip8l1	TNFAIP8L1	TNF alpha induced protein 8 like 1	Cytoplasm	other	
2.22E-02	2.38	Rab26	RAB26	RAB26, member RAS oncogene family	Plasma Membrane	enzyme	
1.45E-02	2.38	Elfn2	ELFN2	extracellular leucine rich repeat and fibronectin type III domain containing 2	Extracellular Space	other	
2.52E-02	2.382	Npy	NPY	neuropeptide Y	Plasma Membrane	other	
2.03E-02	2.386	Akr1c12	Akr1c12/Akr	aldo-keto reductase family 1, member C13	Cytoplasm	enzyme	
3.47E-02	2.389	Arhgap32	ARHGAP32	Rho GTPase activating protein 32	Cytoplasm	other	
5.48E-03	2.389	Trerf1	TRERF1	transcriptional regulating factor 1	Nucleus	transcription regulator	
4.68E-02	2.395	Htr1a	HTR1A	5-hydroxytryptamine receptor 1A	Plasma Membrane	G-protein coupled receptor	methysergide, vilazodone, ziprasidone, SLV-314, naluzotan, ergotamine, xaliproden, doxepin, asenapine, chloropromazine, aripiprazole lauroxil, trazodone, naratriptan, brexpiprazole, zolmitriptan, fenfluramine, dihydroergotan
2.84E-02	2.402	Ccdc116	CCDC116	coiled-coil domain containing 116	Extracellular Space	other	
3.15E-02	2.406	Cacnb3	CACNB3	calcium voltage-gated channel auxiliary subunit beta 3	Plasma Membrane	ion channel	verapamil, dexamethasone/rituximab/verapamil, mibefradil, nimodipine, trandolapril/verapamil
1.17E-02	2.408	Krt33b	KRT31	keratin 31	Cytoplasm	other	
4.94E-03	2.448	Kctd4	KCTD4	potassium channel tetramerization domain containing 4	Other	ion channel	
4.22E-02	2.465	Klk8	KLK8	kallikrein related peptidase 8	Extracellular Space	peptidase	
3.52E-03	2.477	Krt27	KRT27	keratin 27	Cytoplasm	other	
1.78E-02	2.483	Ankrd42	ANKRD42	ankyrin repeat domain 42	Nucleus	transcription regulator	
1.67E-02	2.484	Galnt14	GALNT14	polypeptide N-acetylgalactosaminyltransferase 14	Cytoplasm	enzyme	
2.59E-02	2.49	Pkp1	PKP1	plakophilin 1	Plasma Membrane	other	
2.6E-02	2.504	Prkg2	PRKG2	protein kinase cGMP-dependent 2	Cytoplasm	kinase	
1.5E-02	2.557	Apobec4	APOBEC4	apolipoprotein B mRNA editing enzyme catalytic polypeptide like 4	Other	other	
1.13E-02	2.564	Celf3	CELF3	CUGBP Elav-like family member 3	Nucleus	transcription regulator	
7.9E-03	2.585	Sprn	SPRN	shadow of prion protein	Nucleus	other	
3.08E-02	2.6	Lrrc66	LRRC66	leucine rich repeat containing 66	Other	other	
1.98E-02	2.603	Egr4	EGR4	early growth response 4	Nucleus	transcription regulator	
3.49E-03	2.617	Gm7056	Gm7056	predicted gene 7056	Other	other	
4.89E-02	2.619	Homer2	HOMER2	homer scaffold protein 2	Plasma Membrane	other	
4.72E-02	2.636	Chn1	CHN1	chimerin 1	Cytoplasm	other	
3.58E-02	2.649	Vmn1r233	Vmn1r233	vomeronasal 1 receptor 233	Plasma Membrane	G-protein coupled receptor	
6.33E-04	2.657	Ccl17	CCL17	C-C motif chemokine ligand 17	Extracellular Space	cytokine	
9.38E-03	2.673	Kctd16	KCTD16	potassium channel tetramerization domain containing 16	Plasma Membrane	other	
1E-02	2.689	Klf16	Klf16	Kruppel-like transcription factor 16	Nucleus	transcription regulator	
4.25E-02	2.717	Camk2n1	CAMK2N1	calcium/calmodulin dependent protein kinase II inhibitor 1	Plasma Membrane	kinase	
5.77E-03	2.718	Adrb1	ADRB1	adrenoceptor beta 1	Plasma Membrane	G-protein coupled receptor	KUL 7211, bisoprolol, epinephrine/lidocaine, nebivolol/valsartan, dobutamine, pindolol, penbutolol, bisoprolol/hydrochlorothiazide, guanethidine, latanoprost/timolol, dipivefrin, pirbuterol, guanadrel, hydrochlorothiazide/meto
4.16E-02	2.747	Ccno	CCNO	cyclin O	Nucleus	other	
9.89E-03	2.769	Poc1a	POC1A	POC1 centriolar protein A	Cytoplasm	peptidase	
4.26E-04	2.789	Hkdc1	HKDC1	hexokinase domain containing 1	Cytoplasm	kinase	
1.81E-03	2.833	Zdhhc8	ZDHHC8	zinc finger DHHC-type palmitoyltransferase 8	Cytoplasm	enzyme	
4.61E-02	2.845	Klhl31	KLHL31	kelch like family member 31	Other	other	
1.11E-02	2.884	En1	EN1	engrailed homeobox 1	Nucleus	transcription regulator	
1.33E-02	2.91	Nxph2	NXPH2	neurexophilin 2	Extracellular Space	other	
1.53E-02	2.957	Dazl	DAZ2	deleted in azoospermia 2	Cytoplasm	translation regulator	

**3.66E-03**	2.99	Npas2	NPAS2	neuronal PAS domain protein 2	Nucleus	transcription regulator	
**6.75E-04**	2.991	Plk2	PLK2	polo like kinase 2	Nucleus	kinase	PLK2 inhibitor 37
**1.27E-02**	3.03	Klra5	Klra4 (includ	killer cell lectin-like receptor, subfamily A, member 4	Plasma Membrane	transmembrane receptor	
**9.84E-03**	3.04	Cx3cl1	CX3CL1	C-X3-C motif chemokine ligand 1	Extracellular Space	cytokine	
**5.5E-03**	3.055	Il1rapl2	IL1RAPL2	interleukin 1 receptor accessory protein like 2	Plasma Membrane	transmembrane receptor	
**3.96E-02**	3.058	BC048562	C3orf84	chromosome 3 open reading frame 84	Other	other	
**4.74E-03**	3.094	Fank1	FANK1	fibronectin type III and ankyrin repeat domains 1	Nucleus	transcription regulator	
**5.57E-04**	3.12	Aox4	Aox4	aldehyde oxidase 4	Cytoplasm	enzyme	
**2.89E-02**	3.141	Prm1	Prm1	protamine 1	Nucleus	other	
**9.03E-03**	3.156	Nppa	NPPA	natriuretic peptide A	Extracellular Space	other	
**3.48E-02**	3.171	Gabra5	GABRA5	gamma-aminobutyric acid type A receptor subunit alpha5	Plasma Membrane	ion channel	flunitrazepam, sevoflurane, alprazolam, butobarbital, atropine/hyoscyamine/phenobarbital/scopolamine, phenobarbital, diazepam, barbital, acetaminophen/butalbital/caffeine/codeine, hyoscyamine/phenobarbital, SEP 17455
**3.62E-02**	3.193	Defb19	DEFB119	defensin beta 119	Extracellular Space	other	
**4.65E-03**	3.193	Kcnip2	KCNIP2	potassium voltage-gated channel interacting protein 2	Plasma Membrane	transporter	
**3.19E-02**	3.202	Htr6	HTR6	5-hydroxytryptamine receptor 6	Plasma Membrane	G-protein coupled receptor	iloperidone, sertindole, WAY-181187, olanzapine, asenapine
**4.33E-02**	3.208	Dact2	DACT2	dishevelled binding antagonist of beta catenin 2	Cytoplasm	other	
**2.59E-02**	3.275	Treml1	TREML1	triggering receptor expressed on myeloid cells like 1	Plasma Membrane	other	
**3.75E-02**	3.296	Prok2	PROK2	prokineticin 2	Extracellular Space	other	
**1.21E-02**	3.318	Cpa6	CPA6	carboxypeptidase A6	Extracellular Space	peptidase	
**2.83E-03**	3.368	Fam163b	FAM163B	family with sequence similarity 163 member B	Other	other	
**2.13E-02**	3.388	Glt8d2	GLT8D2	glycosyltransferase 8 domain containing 2	Other	enzyme	
**3.84E-02**	3.395	Chsy3	CHSY3	chondroitin sulfate synthase 3	Cytoplasm	enzyme	
**1.73E-02**	3.427	A830018L16Rik	C8orf34	chromosome 8 open reading frame 34	Other	other	
**1.93E-02**	3.487	Arpp21	ARPP21	cAMP regulated phosphoprotein 21	Cytoplasm	other	
**1.32E-02**	3.491	Fhl2	FHL2	four and a half LIM domains 2	Nucleus	transcription regulator	
**3.66E-02**	3.497	Syt17	SYT17	synaptotagmin 17	Plasma Membrane	other	
**8.61E-03**	3.549	Ak5	AK5	adenylate kinase 5	Cytoplasm	kinase	
**3.88E-02**	3.552	Hes5	HES5	hes family bHLH transcription factor 5	Nucleus	transcription regulator	
**4.37E-02**	3.553	Cyp2c55	CYP2C18	cytochrome P450 family 2 subfamily C member 18	Cytoplasm	enzyme	
**2.44E-02**	3.608	Adamtsl2	ADAMTSL2	ADAMTS like 2	Extracellular Space	other	
**6.93E-03**	3.632	Cpne5	CPNE5	copine 5	Plasma Membrane	other	
**4.85E-03**	3.676	Lzts1	LZTS1	leucine zipper tumor suppressor 1	Nucleus	other	
**4.85E-02**	3.699	Dlx2	DLX2	distal-less homeobox 2	Nucleus	transcription regulator	
**3.87E-02**	3.73	Ankrd33b	ANKRD33B	ankyrin repeat domain 33B	Other	other	
**4.07E-02**	3.787	Trim42	TRIM42	tripartite motif containing 42	Other	other	
**4.43E-02**	3.86	Nptx2	NPTX2	neuronal pentraxin 2	Extracellular Space	other	
**2.16E-02**	3.912	Hs3st2	HS3ST2	heparan sulfate-glucosamine 3-sulfotransferase 2	Cytoplasm	enzyme	
**3.63E-03**	3.919	Lmo3	Lmo3	LIM domain only 3	Cytoplasm	other	
**1.44E-02**	3.931	Arhgap33	ARHGAP33	Rho GTPase activating protein 33	Plasma Membrane	transporter	
**4.67E-02**	3.959	Baiap2l1	BAIAP2L1	BAR/IMD domain containing adaptor protein 2 like 1	Cytoplasm	other	
**1.44E-05**	3.976	Enc1	ENC1	ectodermal-neural cortex 1	Nucleus	peptidase	
**1.18E-02**	3.979	Flg2	Flg2	filaggrin family member 2	Cytoplasm	other	
**1.9E-03**	3.994	Myo5b	MYO5B	myosin VB	Cytoplasm	enzyme	
**2.24E-03**	4.05	Gpr151	GPR151	G protein-coupled receptor 151	Plasma Membrane	G-protein coupled receptor	
**4.58E-02**	4.069	Rprm	RPRM	reprimo, TP53 dependent G2 arrest mediator homolog	Cytoplasm	other	
**8.66E-03**	4.086	Akap5	AKAP5	A-kinase anchoring protein 5	Plasma Membrane	other	
**2.55E-02**	4.136	Tnnc1	TNNC1	troponin C1, slow skeletal and cardiac type	Cytoplasm	other	levosimendan, Ca2+
**4.37E-02**	4.143	Rspo2	RSPO2	R-spondin 2	Extracellular Space	other	
**3.99E-02**	4.149	Cplx3	CPLX3	complexin 3	Nucleus	transporter	
**2.98E-02**	4.176	Prss12	PRSS12	serine protease 12	Extracellular Space	peptidase	
**9.62E-04**	4.378	Sag	SAG	S-antigen visual arrestin	Cytoplasm	other	
**2.94E-02**	4.44	Dlx5	DLX5	distal-less homeobox 5	Nucleus	transcription regulator	
**3.88E-03**	4.481	Mei1	MEI1	meiotic double-stranded break formation protein 1	Other	other	
**3.19E-02**	4.611	Klra19	Klra19	killer cell lectin-like receptor, subfamily A, member 19	Plasma Membrane	other	
**1.18E-02**	4.693	Crym	CRYM	crystallin mu	Cytoplasm	enzyme	
**4.85E-04**	5.297	Dkkl1	DKKL1	dickkopf like acrosomal protein 1	Extracellular Space	other	
**2.94E-02**	5.303	Ctxn1	CTXN1	cortexin 1	Other	other	
**3.62E-03**	5.448	Zfp831	ZNF831	zinc finger protein 831	Other	other	
**1.25E-02**	5.544	Slc26a4	SLC26A4	solute carrier family 26 member 4	Plasma Membrane	transporter	
**2.35E-02**	5.574	Rhcg	RHCG	Rh family C glycoprotein	Plasma Membrane	transporter	
**2.5E-02**	5.653	Trbv1	Trbv1	T cell receptor beta, variable 1	Other	other	
**1.42E-02**	5.696	Dlx6os1	Dlx6os1	distal-less homeobox 6, opposite strand 1	Nucleus	other	
**9.43E-03**	6.463	Kcnj4	KCNJ4	potassium inwardly rectifying channel subfamily J member 4	Plasma Membrane	ion channel	
**2.09E-02**	6.569	Icam5	ICAM5	intercellular adhesion molecule 5	Plasma Membrane	other	
**2.56E-02**	6.62	Rnf148	RNF148	ring finger protein 148	Extracellular Space	enzyme	
**1.94E-03**	6.69	Rasl10a	RASL10A	RAS like family 10 member A	Plasma Membrane	enzyme	
**3.36E-02**	7.307	Ddn	DDN	dendrin	Cytoplasm	transcription regulator	

H1-Hallmark Database Top 20 Gene Sets	Size	ES	NES	FDR q-value
HALLMARK_INTERFERON_GAMMA_RESPONSE	102	0.6	1.6	0.144
HALLMARK_ALLOGRAFT_REJECTION	110	0.51	1.6	0.095
HALLMARK_INFLAMMATORY_RESPONSE	98	0.54	1.5	0.279
HALLMARK_WNT_BETA_CATENIN_SIGNALING	22	0.55	1.42	0.286
HALLMARK_IL6_JAK_STAT3_SIGNALING	49	0.58	1.41	0.238
HALLMARK_KRAS_SIGNALING_DN	90	0.41	1.39	0.325
HALLMARK_INTERFERON_ALPHA_RESPONSE	37	0.6	1.36	0.334
HALLMARK_TNFA_SIGNALING_VIA_NFKB	110	0.49	1.36	0.319
HALLMARK_KRAS_SIGNALING_UP	101	0.43	1.26	0.514
HALLMARK_CHOLESTEROL_HOMEOSTASIS	38	0.49	1.23	0.562
HALLMARK_COMPLEMENT	93	0.41	1.21	0.594
HALLMARK_IL2_STAT5_SIGNALING	94	0.43	1.2	0.549
HALLMARK_APOPTOSIS	86	0.41	1.2	0.514
HALLMARK_PI3K_AKT_MTOR_SIGNALING	55	0.39	1.16	0.582
HALLMARK_MYC_TARGETS_V2	25	0.38	1.16	0.546
HALLMARK_PANCREAS_BETA_CELLS	23	0.42	1.13	0.594
HALLMARK_MTORC1_SIGNALING	98	0.3	1.12	0.58
HALLMARK_ESTROGEN_RESPONSE_EARLY	82	0.36	1.11	0.607
HALLMARK_APICAL_JUNCTION	91	0.35	1.1	0.593
HALLMARK_MYOGENESIS	100	0.32	1.09	0.621

Significant Gene Set (FDR < 25%)

Significant Immune-Related Gene Set (FDR < 25%)

Immune-Related Gene Set (FDR > 25%)

Supplementary Table S2. Top 20 H1-Hallmark database gene sets identified by GSEA in the pre-symptomatic *Npc1*$^{-/-}$ cerebral cortex. ES = enriched score; NES = normalized enriched score; FDR = False discovery rate q value

	Npc1$^{-/-}$ vs. wildtype			
	Cerebellum (CRB2)		Cerebral Cortex (CTX2)	
Cytokine	FC	p-value	FC	p-value
Ccl2	3.286	0.026	ns	ns
Ccl4	ns	ns	4.664	0.0489
Ccl5	4.772	0.0152	ns	ns
Ccl6	4.237	0.0134	ns	ns
Ccl7	1.961	0.0299	ns	ns
Ccl9	ns	ns	1.624	0.039
Ccl21	ns	ns	2.823	0.0105
Cklf	ns	ns	1.809	0.03
Csf1	ns	ns	2.183	0.0139
Cxcl3	ns	ns	1.812	0.0386
Cxcl6	1.754	0.0413	ns	ns
Cxcl10	11.722	0.017	ns	ns
Cxcl12	ns	ns	1.984	0.0276
Ebi3	ns	ns	2.101	0.007
Il9	ns	ns	1.964	0.0088
Spred2	ns	ns	1.862	0.0221
Ccl24	-3.755	0.035	-2.887	0.0492
Ccl28	ns	ns	-1.514	0.0287
Clcf1	-2.152	0.0017	ns	ns
Cmtm5	ns	ns	-1.696	0.0491
Il23a	ns	ns	-2.746	0.0139

Supplementary Table S3. Differential expression of cytokine transcripts in the cerebral cortex vs. the cerebellum in the *Npc1*$^{-/-}$ mice
